# Transition Metal Dichalcogenide MoS_2_: Oxygen and Fluorine Functionalization for Selective Plasma Processing

**DOI:** 10.1021/acs.jpclett.6c00348

**Published:** 2026-04-27

**Authors:** Yury Polyachenko, Yuri Barsukov, Shoaib Khalid, Igor Kaganovich

**Affiliations:** † 6740 Princeton Plasma Physics Laboratory, Princeton, New Jersey 08540, United States; ‡ Department of Chemistry, 17217 Princeton University, Princeton, New Jersey 08544, United States

## Abstract

Low-temperature plasma processing is a promising technique for tailoring transition metal dichalcogenides (TMDs). For chalcogen substitution processing, a key challenge is to identify the ion energy window that enables selective chalcogen removal while preserving the metal lattice. Using ab initio molecular dynamics (AIMD), we demonstrate that oxygen and fluorine functionalization widen the processing window by significantly lowering the sulfur sputtering energy threshold (*E*_sputt,S_) of MoS_2_ from ∼30 to ∼10 eV via formation of sputtering products such as SO_2_ and SF_*n*_. Additionally, we show that experimentally relevant cryogenic temperatures strongly affect *E*_sputt,S_(*T*) . The dependence is confirmed via AIMD and also predicted by a mechanistic parameter-free theory, suggesting that *E*_sputt_ (*T*) generalizes to other TMDs, functionalizations, and surface impact conditions. Our results highlight oxygen/fluorine functionalization, ionic impact angle, and material temperature to be key control parameters for selective, damage-controlled chalcogen removal in TMD processing.

## Graphical abstract

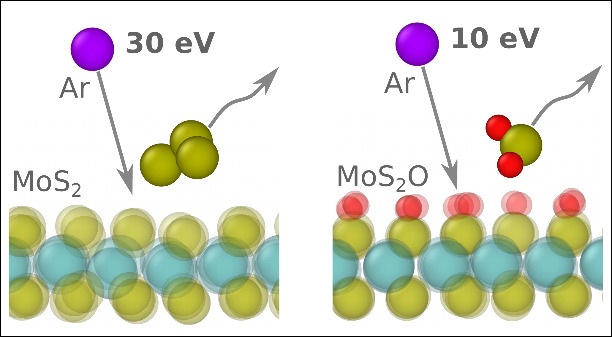

Plasma processing offers a powerful technique for tailoring material structure and properties.^1^ Plasma processing has been widely applied to two-dimensional (2D) materials, including graphene^2–4^ and transition metal dichalcogenides (TMDs).^5–11^ For TMD processing, ion bombardment underpins a variety of processing steps such as etching, cleaning, and doping^12^ where selective removal of targeted atoms is required while preserving the lattice structure. Among TMDs, monolayer MoS_2_ in its 2H phase stands out as a particularly promising candidate owing to its direct band gap, comparable to silicon, but at a much smaller thickness.^13^ A critical factor in plasma processing of MoS_2_ is identifying the ion energy window that enables selective chalcogen removal while preserving the metal lattice.

Pristine MoS_2_ has been widely studied through both experimental and theoretical approaches: precise ionic bombardment,^14–20^ excitations,^21–27^ multicharged ions,^28–31^ ion neutralization,^32–34^ substrate effects,^35–37^ strain.^38–41^ Experiments have shown that Ar^+^ ions with kinetic energies of approximately 50 eV are sufficient to generate sulfur vacancies (S vacancies), while energies near 100 eV are required to remove Mo atoms and ultimately etch the entire layer in MoS_2_.^14^ This establishes a baseline energy window for plasma processing. However, modern techniques are capable of creating ions with temperatures as low as *T*_i_ ∼ 1 eV and also applying a bias of ∼10–20 eV.^42,43^ This allows controlling ion energies of 10–20 eV to within ± 1 eV. Therefore, these plasmas can be used to create an ion flux with precisely controlled energies. In this work, we calculate the sputtering threshold energy, *E*_sputt,S_, of MoS_2_ with significantly improved accuracy and propose a novel strategy to reduce this threshold by functionalizing the TMD surface with oxygen or fluorine. Based on ab initio molecular dynamics (AIMD) simulations, we develop a sputtering mechanism that explains the observed results, including a prediction of temperature dependence of *E*_sputt,S_ for oxygen-functionalized MoS_2_.

Previous theoretical studies have estimated that to eject a S atom from MoS_2_, approximately 7 eV of energy directed exactly outward from the material needs to be transferred to the sulfur atom.^21,44^ This value decreases to about 4–6 eV when considering excitations of various valence electrons,^26^ and can be as low as 2.2 eV in the presence of multiple excitations of core electrons, i.e. core–electron excitations. Electron scattering experiments have inferred an effective sulfur desorption energy of approximately 1.5 eV,^22^ suggesting that incident electrons generate multiple deep excitations while transferring momentum to the S atom. Low-energy plasma processing is generally conducted with ion energies not exceeding approximately 100 eV.^12^ Under these conditions, the generation of deep excitations is unlikely, since the sulfur 2*p* peaks in X-ray photoelectron spectroscopy (XPS) spectra of MoS_2_, corresponding to core excitation energies, appear above 150 eV.^45^ Moreover, coupling between electronic and ionic motion is inefficient due to their large mass disparity, which makes fast energy transfer to electrons infeasible and gives the ion time to lose its energy in multiple collisions. Therefore, we estimate that the lower bound of the sulfur escape energy threshold is *E*_escape_ = 6––7 eV.

During TMD-ion collisions, the energy *E*_escape_ is what needs to be transferred to the impacted S atom. This transferred energy is smaller than the energy of the incoming projectile *E*_hit_ due to the mass ratio *m*_S_/*m*_I_ ≠ 1 between the impacted S atom and the ion. Therefore, the lower bound for the sputtering projectile energy *E*_hit_ is larger than the escape energy by the following factor:

1 $$E_{\mathrm{hit}}=E_{\mathrm{escape}}\cdot {\left(\frac{\sqrt{m_{S}/m_{I}}+\sqrt{m_{I}/m_{S}}}{2}\right)}^{2}$$

However, the collisions are not binary and involve other atoms. Thus, the energy transferred from ion to sulfur atoms can be shared with neighboring molybdenum or other sulfur atoms, so the threshold energy for removal can be higher.

Earlier molecular dynamics simulations on 2D materials have demonstrated that when ion energies exceed 100 eV, the sputtering yield is mainly controlled by secondary collisions involving atoms that are reflected or knocked out from the supporting substrate.^36^ However, at lower energies close to the damage threshold (around 10 eV), the sputtering process is expected to be substantially different. The energy barely sufficient to cause a sulfur ejection is not enough to significantly perturb the material below the top TMD layer, especially given relatively wide gaps between TMD layers. Thus, the substrate can be safely excluded from the simulations. Instead, so-called ”chemically enhanced physical sputtering” was described for silicon, when ejecting more chemically stable reaction products rather than individual atoms often reduced the minimum energy required for sputtering.^46^ When an ion collides with the top sulfur atom, the impacted sulfur atom initiates a cascade of intermediate collisions that can affect TMD lattice. Careful consideration needs to be given to these intermediate collisions so that the TMD lattice is restored after the collision and is not damaged.

In this research, we propose a two-step chemically enhanced physical sputtering mechanism that minimizes the sputtering threshold energy *E*_sputt,S_ of MoS_2_ functionalized by oxygen or fluorine:

1. The Ar impact induces atomic rearrangements that facilitate the formation of (meta)­stable gas-phase species such as SO_2_, SF_4_.
2. Under the above condition, efficient momentum transfer from the incoming Ar to the ejected molecular species is achieved by optimizing the number and directions of intermediate collisions that lead to desorption of these products.

The simulation setup for collisions of the incoming Ar with monolayer MoS_2_ is shown in Figure 1.

**Figure 1:**
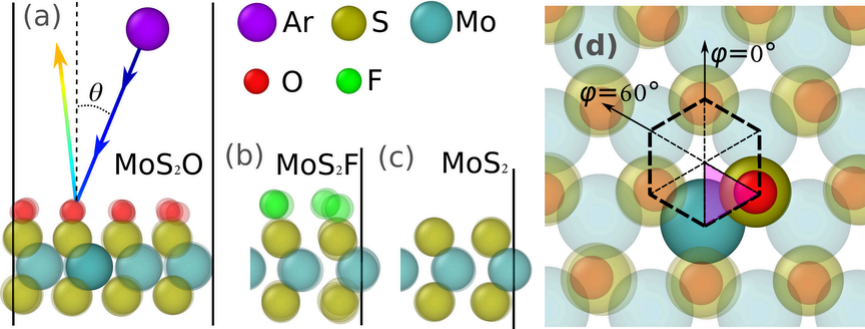
Simulation setup schematic. (a) Side view of the full MoS_2_O equilibrated supercell (4 × 4) initial state. Ar atom’s trajectory is shown by a rainbow line with color representing time from blue to red. (b,c) Representative parts of the MoS_2_F and MoS_2_ initial equilibrated states (side view), respectively. The irregularity of the fluorine atoms’ positions is discussed in the main text. (d) Top view of MoS_2_O. The purple shade triangle shows the minimal set of hit-points that represent all other points by symmetry. The in-plane far-field approach angle, φ, shows its origin φ = 0° and its positive direction φ = 60°. The hit angle remains very close to the initial far-approach angle in cases considered in this study.

In this paper, we show that oxygen or fluorine functionalization reduces the sputtering threshold energy *E*_sputt,S_ in MoS_2_, as illustrated in Figure S2. This phenomenon is explained by the observation that small (meta)­stable molecular fragments, such as SO_2_ and SF_4_, can form more easily from a few TMD atoms actively involved in a low-energy Ar impact, compared to the ejection of pure sulfur atoms or clusters (S_2_, S_6_, or S_8_). The enhanced formation of these O- or F-containing fragments can be attributed to O/F’s high electronegativity, which stabilizes intermediate fragments relative to the rest of the material and facilitates their ejection under lower-energy impacts. These findings suggest that selective functionalization provides a practical route to control the sputtering behavior of different TMDs at energies near the damage threshold.

As a preliminary step, we examined the feasibility of O and F adsorption on MoS_2_ before analyzing the impacts of O and F covered surfaces. It is well-known that oxygen exhibits strong reactivity with TMDs.^47–58^ For example, even molecular O_2_ has been shown to fill S vacancies in MoS_2_ with an activation barrier of approximately 1.05 eV.^48^ Previous calculations have further indicated that O_2_ can dissociate into atomic oxygen, and, subsequently, adsorb onto MoS_2_, with a total barrier of about 1.6 eV.^54^ Isolated atomic oxygen was also reported to adsorb on MoS_2_ with a binding energy of 0.87–1.12 eV, depending on the surface coverage.^54^ Our calculated adsorption energy for O on MoS_2_ is in close agreement with the known results, as shown in Figure S3b of the Supporting Information. Differences of ∼0.1–0.2 eV may have resulted from a smaller unit-cell and k-point sampling, but are deemed acceptable given the studied energy scale of ∼10 eV. Furthermore, we performed meta-dynamics calculations to verify that our conclusions based on the potential energy surfaces (PES) qualitatively hold for the corresponding free-energy surfaces (FES) as shown in Figure S3d,e.

Oxygen has been experimentally shown to play a significant role in the thermal etching of TMDs, initiating around 345 °C.^55^ Under ambient conditions, approximately month-long exposure to molecular O_2_ also induces sulfur vacancies.^56^ In both cases, the formation of stable gas-phase species such as SO_2_ has been proposed as a key step in oxygen-assisted etching. On the other hand, it has been demonstrated that TMDs can undergo oxygen functionalization, which is detectable in characterization techniques such as PL and XPS.^12^ Finally, processing energy windows were investigated.^14^

Despite the damage energy threshold being a key parameter in processing, the interplay between TMD functionalization and changes in the sputtering energy threshold has not yet been quantitatively explored. Inspired by observations above, we show a significant drop in the threshold sputtering energy induced by adsorbed oxygen and fluorine, which can considerably ease chalcogen plasma-assisted removal in TMDs. We quantify how such functionalization could extend the Ar ion energy window for plasma processing and enhance the selective removal of chalcogen atoms while preserving the underlying metal lattice. We also confirmed and explained coupling of the sputtering threshold to the impact angle and to TMD temperature. Reported effects rely on a simple mechanistic theory and are expected to generalize to other TMDs and appropriate functionalizations.

We first investigate impacts orthogonal to the material plane (θ = 0, see Figure 1a). Figure S2 shows the probabilities of sulfur ejection *P*_sputt_ for pristine and functionalized MoS_2_. Atomic oxygen functionalization decreases *E*_sputt,S_ from ∼(31 ± 1) to ∼(14.0 ± 1) eV. Atomic fluorine is even more effective with *E*_sputt,S_ ∼ (9.5 ± 0.5) eV. Full surface coverage is considered in our model. The reduction in the sputtering threshold arises from the formation and desorption of products such as SO_2_ and SF_4_. Thus, using combinatorial arguments about occupation of neighboring sites by oxygen or fluorine, we show that surface coverage *c* ∈(0;1) should decrease the probability of the proposed mechanisms by *c* (details in Supporting Information “Surface coverage fraction effects”). No back-sputtering of Mo atoms is observed up to *E*_Ar_ = 50 eV for MoS_2_ and the layer destruction is seen at 100 eV, confirming the experimentally known constraint.^14^ No Mo back-sputtering is seen below 50 eV for MoS_2_O and MoS_2_F, so the processing energy window indeed seems to be widened by O/F functionalization.

These values correspond to the energy of an Ar projectile that results in sulfur sputtering at least once out of *N*_hits_ given the most susceptible hit-point (detailed in Figure S4). At least *N*_hits_ ≥ 14 was used in all reported simulations. This describes sampling at a fixed hit-point, and we estimate it to correspond to sampling ∼4000 impacts per elementary triangle (see the Supporting Information). The threshold definition technically depends on *N*_hits_, which is expected since any event can be observed once given sufficient sampling. However, we estimate (details in the Supporting Information) that the dependence is weak $∼\sqrt{\ln\left(N_{\mathrm{hits}}\right)}$ . We also estimate a lower bound for sputtering yield near the computed energy threshold *Y*_*S*_ ≥ 0.0036·*P*_sputt_ (detailed in the Supporting Information), which is approximately consistent with force-field MD results for MoS_2_.^36^ Additionally, sputtering requires defining the time scale of ejection because weakly adsorbed products such as SO_2_ can take a long time to desorb. These effects set an energy range ∼1 eV wide near the energy threshold, where increasing observation time meaningfully decreases the calculated energy threshold. Based on representative longer AIMD (Figure S7), we choose 2 ps as the cutoff time that optimally separates unimpeded ejections from thermal product desorption. Finally, the determination of the damage energy threshold instead of the direct yield requires only the simulation of impacts of damage-free material because we only quantify the transition from no-damage to some-damage. This allows us to avoid relatively long simulations of many consecutive impacts of the same cell and instead run many independent single impacts in parallel.

The energy error bars represent the spacing of the energy grid used to determine the sputtering threshold because statistical errors were found to be much smaller. The precise threshold values can change depending on the specific approximations used in DFT, such as the selected functional or pseudopotential. For this reason, we did not further refine the energy grid. We demonstrate in the Supporting Information that the results are sufficiently insensitive to the choice of DFT functional, dispersion correction scheme, k-point sampling, energy cutoff, and other computational settings. Because our study focuses on oxygen, we explicitly examine the relevant spin states. Oxygen is shown to have a singlet ground-state when bound to TMDs (Figure S3b), and the Supporting Information outlines our reasons for running all collision dynamics in fixed singlet spin state. For completeness, the triplet is shown to qualitatively preserve the significant drop in the sputtering energy threshold *E*_sputt,S_ from O/F functionalization. We note that the qualitative relative effect of the substantial reduction in *E*_sputt,S_ is expected to be insensitive to most of the DFT details, as it arises from clear physical mechanisms discussed below.

These results suggest that O and F prefunctionalization can effectively expand the ionic energy window for plasma processing, allowing selective chalcogen removal at lower projectile energies, while minimizing damage to the underlying metal scaffold.

A qualitative understanding of why the threshold drops so significantly can be obtained by examining ejection events that are representative of MoS_2_O and MoS_2_F, as shown in Figure 3 and Figure S1.

**Figure 2:**
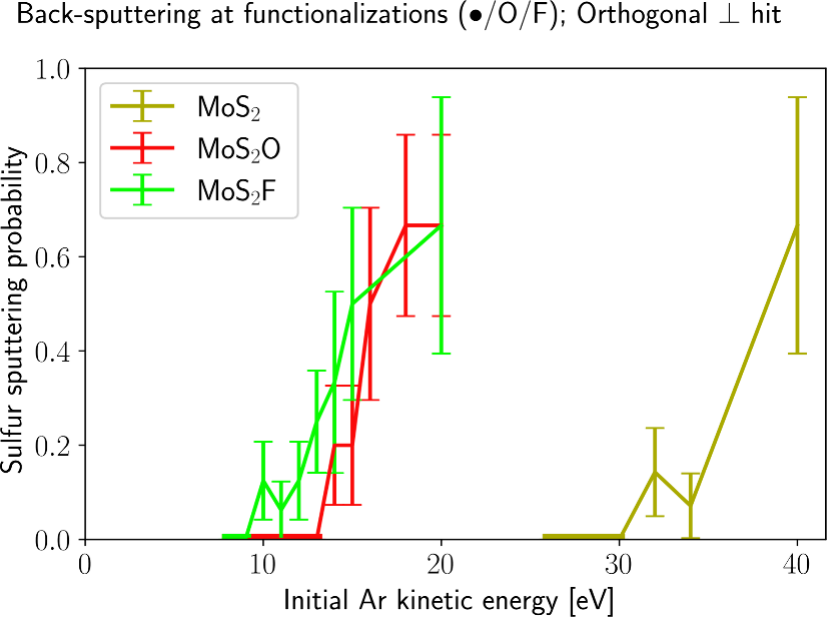
Probability to eject sulfur from pristine and functionalized MoS_2_ back into the half-space the impact came from. Functionalization with O and F substantially lowers the sputtering threshold energy, indicating enhanced chalcogen removal efficiency at reduced Ar energies. For each of three TMDs, the impact point was optimized to minimize *E*_sputt,S_ (details in Figure S4). Materials were equilibrated at 116 K.

**Figure 3:**
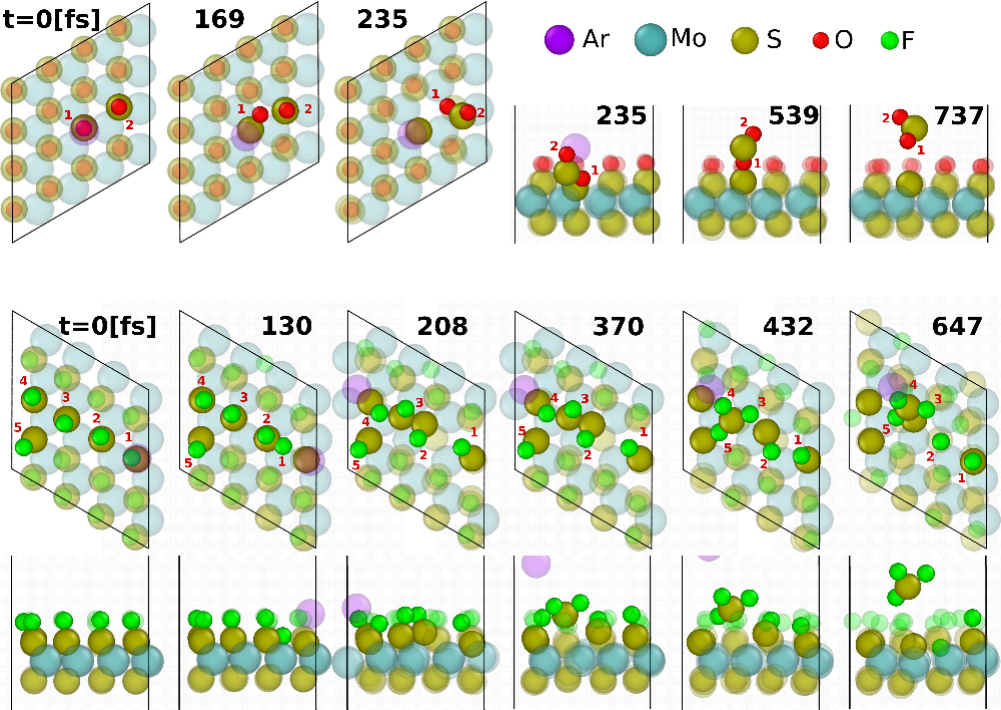
(Top) A typical orthogonal Ar collision with MoS_2_O. Opaque atoms are those that ultimately play a major role in the collision. Gray numbers are timestamps in [fs]. The sputtering mechanism of MoS_2_O that realizes at the lowest required projectile energy involves an O atom being pushed in the direction of the nearest S atom that already has another O bound to it, allowing them to combine and form SO_2_, which then escapes. Small red numbers enumerate O atoms to help follow them during collisions. A collision at an angle is shown in video V1. (Middle and Bottom) A typical orthogonal Ar collision with MoS_2_F. Side and top views are both provided to illustrate more complex atom movements during the collisions. Small red numbers enumerate F atoms to help follow them during collisions. Both materials were equilibrated at 116 K, both impacts were with 15 eV.

A typical collision that results in sulfur sputtering from an O- or F-functionalized MoS_2_ involves the formation of (meta)­stable products (such as SO_2_ or SF_4_) that later escape. This is different from pristine MoS_2_ sputtering (details are provided in Figure S1), which first mechanically breaks bonds of S atoms that later may assemble into S_n_ cluster products. Accordingly, we ascribe the substantial variation in sputtering energy thresholds to the distinct sputtering mechanisms involved.

A noticeable difference in typical collision events between MoS_2_O and MoS_2_F is attributed to the differences in equilibrium positions of adsorbed O and F atoms on the TMD surface, shown in Figure 3 (at *t* = 0). More details are discussed in the Supporting Information.

The MoS_2_O structure keeps all symmetries of MoS_2_ and oxygen atoms are adsorbed directly above the sulfur atoms (see Figure 3 (*t* = 0) and Figure S5). In contrast, MoS_2_F has a more disordered F layer (see Figure 3 at *t* = 0 and Figure S6). We believe this is analogous to “Peierls distortions” ^59–62^ due to odd number of electrons brought to a unit cell by a fluorine atom (see the Supporting Information for more discussion). This leads to more chaotic collision dynamics. This is indicated by 5 F atoms significantly participating in the MoS_2_F impact, as opposed to only 2 O atoms for the MoS_2_O impact. Sputtering probabilities as a function of the ejected species for conditions of Figure 2 are shown in Figure S2. It suggests that sputtering products from MoS_2_F are more varied than those from MoS_2_O, which further indicates more disorder in the MoS_2_F case. A similar diversity of SF_*n*_ products was reported before for F_2_ plasma.^63^

The observed difference in MoS_2_O and MoS_2_F ground states may be utilized for high-specificity etching due to the following: Because MoS_2_O preserves the well-structured MoS_2_ lattice, collision results depend strongly on Ar incident angle. Correspondingly, a strong angular dependence in the sputtering threshold was found for MoS_2_O and MoS_2_ as shown in Figure 4.

**Figure 4:**
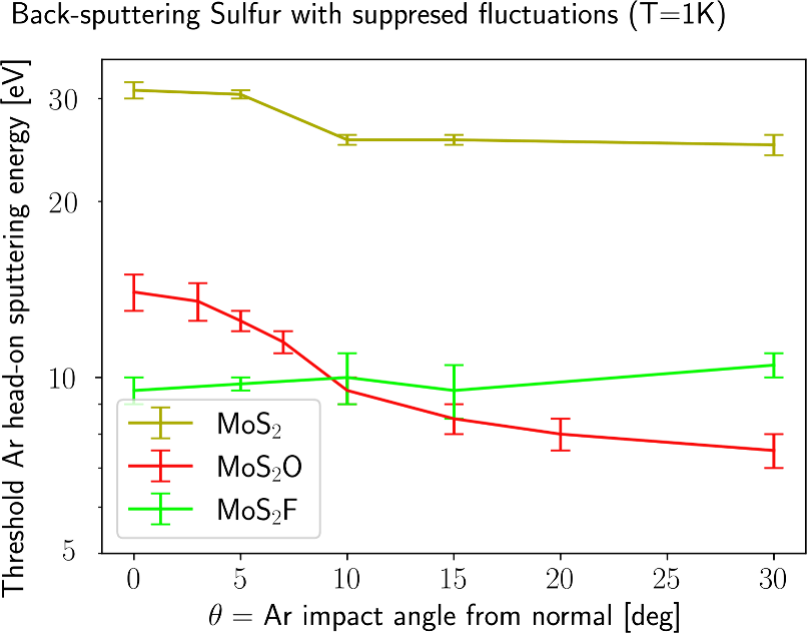
Angular dependence of the sputtering threshold energy *E*_sputt,S_ for MoS_2_ (dark yellow), MoS_2_F (light green) and MoS_2_O (red) for head-on impacts. Thermal fluctuations of target S/F/O atoms in the system are suppressed by formally setting *T* = 1 K. Each curve is obtained after optimizing the impact point and the in-plane angle φ to minimize *E*_sputt,S_, as shown in Figure S10 for MoS_2_O. Simulations at θ = 45° were done for MoS_2_ and MoS_2_O and they showed *E*_MoS_2__(45°) > 35 eV and *E*_MoS_2_O_(45°) > 14 eV. Error bars reflect the step of the energy grid used to pin down the threshold.

The threshold decreases significantly and nonmonotonically with increasing impact angle, reaching about (7.5 ± 0.5) eV = *E*_sputt,S_(θ = 30°) ≈ 0.54·*E*_sputt,S_(θ = 0°) for the MoS_2_O. The value *E*_sputt,S_(θ = 45°) (not shown on the plot) is >14 eV, so θ ≈ 30° is optimal for sputtering MoS_2_O. A similar decrease by ∼6–7 eV is seen for pristine MoS_2_, which also has a clear periodic structure before the impact.

These three presented TMD cases demonstrate a correlation between the degree of order in a system’s ground state and its sensitivity to the angle of projectile impacts. Specifically, sufficiently disordered systems such as MoS_2_F lose their sensitivity to impact direction.

To solidify our understanding of the proposed sputtering mechanisms, we formulate a simple theory that predicts the dependence of the sputtering threshold *E*_sputt,S_ on the TMD temperature *T*. The theory does not require MD simulations at different temperatures, but uses only the temperature-independent curve *E*_sputt,S_(θ) from Figure 4 and simple collision models (see the Supporting Information). We show below that such a simple and parameter-free theory is well correlated with MD simulation results, suggesting the proposed simple mechanisms indeed take place.

The proposed theory is based on connecting the angular dependence *E*_sputt,S_(θ) and thermal fluctuations of target atoms, which cause an unavoidable spread in deflection angles. Because the magnitude of thermal fluctuations of target O/F atoms in the material (about 0.15 Å, Figure S9) is comparable to the distances at which Ar interacts strongly with the O/F atoms (i.e., hard-core repulsion interaction distance, about 1 Å, see Figure S9), a certain range of deflection angles, θ_*T*_, often occurs due to thermal fluctuations. On the other hand, each Ar impact energy *E*_Ar⊥_ has a threshold angle θ_thr_(*E*_Ar⊥_) such that impacts at larger angles θ > θ_thr_(*E*_Ar⊥_) result in sputtering. This means that if the thermal spread of deflection angles θ_*T*_ covers θ_thr_ for a given temperature, i.e. θ_*T*_(*E*_Ar⊥_) > θ_thr_(*E*_Ar⊥_), then deflection angles sufficient for sputtering at the given *E*_Ar⊥_ will be realized frequently, so sputtering will occur frequently. This is quantitatively explained in the SI in Figure S8. Quantification of this logic is given in the Supporting Information, and it yields the oxygen lines in Figure 5.

**Figure 5:**
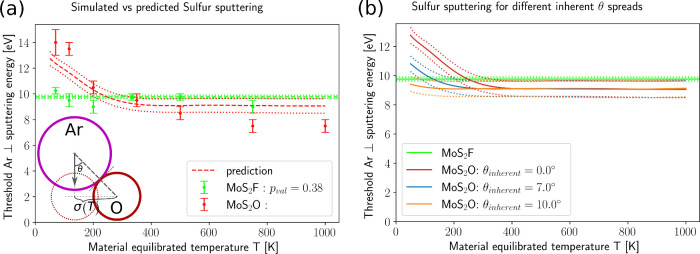
(a) AIMD simulation threshold (data points) at different temperatures for θ = 0 and their predictions (dashed: mean, dotted: confidence interval) based on *E*_Ar⊥_(*T*) from eq S6. Red and green correspond to MoS_2_O and MoS_2_F, respectively. No fitting parameters were used (with the exception of the spline parameters used for interpolating spline data of *E*_Ar–X_(θ) from Figure 4). (a-inset) A schematic of an Ar impacting an O atom (red solid circle) that thermally fluctuated from its equilibrium position (red dashed circle). The Ar velocity is directed exactly at the O equilibrium position, which was shown to be the most damage-susceptible point of MoS_2_O in Figure S4. However, the impact is not head-on due to a thermal fluctuation of magnitude σ­(*T*) of the O atom. (b) Red, blue and yellow: Our predictions for *E*_⊥, sputt_(*T*) of MoS_2_O for inherent θ-spreads of the incoming Ar of 0°, 7°, and 10° respectively. Green: The *T*-independent *E*_sputt,S_ of MoS_2_F. Dotted lines in (a) and (b) show confidence intervals based on error bars in Figure 4.

Notably, this is a parameter-free theory in the sense that no fitting to AIMD *E*_sputt,S_(*T*) was done to obtain red dashed theory line in Figure 5a. Still, the predicted behavior is confirmed in AIMD simulations. It suggests the proposed sputtering mechanism and the theory that follows from it are reasonable and consistent approximations that provide insights into atomistic mechanisms of sputtering of MoS_2_ and its functionalized modifications. Therefore, the proposed sputtering picture can inform TMD simulations at the continuum level and potentially explain the nonlinearities associated with oxygen involvement.^64^

Our analysis shows how functionalization of MoS_2_ with O or F can significantly lower the sputtering energy threshold from ∼30 eV down to ∼10 eV, as shown in Figure S2. Such functionalization may be used for TMD processing with minimal damage to the metal scaffold. Additionally, this type of functionalization enables spatial control over the etching process, which can be realized by selectively depositing oxygen or fluorine in accordance with a mask. Employing partial coverage with oxygen/fluorine can allow accurate control over the creation of sulfur vacancies by selectively sputtering a defined fraction of surface atoms that have been functionalized with oxygen/fluorine, and this approach is relatively insensitive to the exact plasma exposure time.

The main mechanism responsible for the reduction in the energy threshold is attributed to the formation and subsequent desorption of products such as SO_2_ and SF_*n*_. A fully quantitative investigation is required to establish the limits of generalization of this mechanism; however, we expect similar behavior to occur in other TMDs and for other functionalizations. Preliminary AIMD simulations for MoSe_2_, WS_2_, and WSe_2_ with 100% O and F termination indicate that the formation of species such as SF_3_, SO_2_, SeF_3_, and SeO_2_ dominates the sputtering pathways near the threshold energies. Functionalization with O or F similarly reduces the sputtering threshold by approximately a factor of 3, from ∼30 to ∼10 eV. In the case of oxygen termination, the hexagonal structure is largely preserved and the system retains sensitivity to the impact direction. In contrast, fluorine termination breaks the hexagonal symmetry, as observed for MoS_2_F, and the directional dependence is largely lost. Substitution of W for Mo does not lead to significant differences in the threshold energy within the uncertainty of ±3 eV.

A similar argument is expected to hold for most functionalizing atoms, except for very light species such as hydrogen. Because hydrogen is much lighter, it is less effective at slowing down incoming Ar atoms, so the resulting collision pathways are likely to differ from those involving O or F. In addition, hydrogen adsorption is more complex, likely due to its small size. Atomic hydrogen can occupy several competing adsorption sites, including an interstitial site in the plane of the metal (Mo) atom inside the hexagonal lattice.^65,66^ Consequently, more detailed studies are required to quantitatively assess sputtering in H functionalized TMDs such as MoS_2_H.

More specifically, MoS_2_ and MoS_2_O exhibit ranges of possible threshold energies: 32–24 eV and 14–7.5 eV, respectively, which depend on the impact angle (Figure 4). However, achieving the threshold energies of ∼(32 ± 1) eV for MoS_2_ and ∼(14 ± 1) eV for MoS_2_O under strictly normal Ar incidence is likely challenging under realistic plasma conditions (Figure 5b, θ_inherent_ > 7°), where projectiles exhibit an angular spread. In practice, the effective threshold values will be lower because temperature effects disrupt perfectly orthogonal impacts. First, plasma ions have a transverse temperature of at least ∼0.1 eV, which creates a spread in impact angles ∼5° even under low-pressure conditions.^67^ Second, even if some ions impinge on the TMD surface in a truly orthogonal direction and hit O atoms, the O atoms deflect with an angular spread θ_*T*_ (Figure 5b, θ_inherent_ = 0). This spread, θ_*T*_, is determined by the TMD temperature, because thermal fluctuations of the target O atoms grow with increasing temperature, roughly following a ∼√*T* scaling. Therefore, the thresholds observed in experiments are more likely to be closer to ≈(26 ± 2) eV for MoS_2_ and ∼(9 ± 1) eV for MoS_2_O. By tightly restricting the Ar incidence angle to within a spread of <5° and minimizing thermal fluctuations by cooling the TMD to cryogenic temperatures of approximately (−200 to −50 °C, it may be possible to increase the threshold closer to ∼(31 ± 2) eV for MoS_2_ and ∼(13 ± 1) eV for MoS_2_O. To observe the proposed temperature sensitivity, one can also use narrow beams with a very small angular divergence.

One should also keep in mind the limitations of the reported findings. First, the precise numerical values of the reported energy thresholds can depend on the specific details employed in the DFT calculations. However, the key conclusions that sputtering is substantially enhanced by oxygen/fluorine functionalization (and also by increased impact angle) follow from straightforward physical arguments, indicating that they should remain valid regardless of the specific DFT implementation. Second, the proposed sputtering mechanism where products such as SO_2_ and SF_*n*_ form and then desorb requires a high surface coverage of oxygen and fluorine (∼10% or higher). Such coverages are reasonable, given the strong adsorption of atomic oxygen on MoS_2_ ^54^ and other TMDs such as WS_2_.^68^ However, substitutions of S atoms with O atoms in the TMD itself are also favorable in certain cases,^49^ so kinetic tuning may be required to reach high adsorption coverage without significant TMD structure changes. Achieving the necessary surface coverage can be accomplished with fast gas valves and low energy plasma sources.^69^ Cleaning the functionalizing O/F atoms after achieving the desired processing effect also requires additional steps such as high-vacuum annealing.^53^ The removal of residual functionalizing atoms is essential to preserve downstream device quality, motivating further systematic studies of postprocessing cleaning approaches. Additionally, the stability of the system in the presence of chemically reactive gases, such as SF_6_ used during functionalization, warrants further investigation. Notably, a similarly reactive gas, MoF_6_, has been employed in atomic-layer etching^70^ and was observed to induce damage only when alternated with another reactant, such as H_2_O. Furthermore, at low adsorption coverage, different mechanisms are expected, since the formation of SO_2_ and SF_*n*_ under these conditions is unlikely. Similarly, defects from growth stage or grain boundaries may favor different sputtering pathways with low energy threshold, but this goes beyond the scope of this work. Finally, a realistic ion angular spread of about 5–10°^67^ would decrease the temperature sensitivity, as shown in Figure 5b. More MD simulations may help to quantify such effects.

## Supplementary Material







## Notes

Sputtering yield estimates at near-threshold energies, Estimations of effects of surface coverage < 100 % on yeild, Chemical and physical origins of differences of sputtering mechanisms for MoS_2_O vs MoS_2_F, Explanation of interconnection between angular sensitivity and ground-state order, Finding the most damage-susceptible hit point, Simple theory for MoS_2_O *E*_sputt_(*T*), 2-body collision model, Derivation details, High and low *T* regimes of *E*_sputt_(*T*), Spin treatment, DFT applicability, DFT and AIMD details, Free-energy simulation setup; Figures of typical sputtering, adsorption, most damage-susceptible point, equilibration before impact, geometry optimization, AIMD, angular dependence, in-plane thermal fluctuations, in-plane impact angle, Ar–O collision schematic (PDF)
Transparent Peer Review report available (PDF)
(V1) Impact of a single 10 eV Ar at a MoS2O single-layer TMD equilibrated at 350 K at an angle. It results in formation and sputtering of SO_2_, demonstrating the ”chemically enhanced physical sputtering” mechanism. (V2) Impact of a single 34 eV Ar at a MoS_2_ single-layer TMD equilibrated at 350 K normally. It results in “reflection” of Ar from the Mo layer, followed by formation and sputtering of S3. It demonstrates the dominant sputtering pathway at low-energy sputtering. (ZIP)
