# Supplementary material for: Transition Metal Dichalcogenide MoS2: Oxygen and Fluorine Functionalization for Selective Plasma Processing
Source: J Phys Chem Lett. 2026 Apr 27;17(18):5207–14. doi: 10.1021/acs.jpclett.6c00348 (PMC13158984; doi:10.1021/acs.jpclett.6c00348)
Supplement: Supplementary file 1 [file jz6c00348_si_001.pdf]

Transition Metal Dichalcogenide MoS_2: Oxygen and Fluorine Functionalization for Selective Plasma Processing

Yury Polyachenko,*,†,‡ Yuri Barsukov,† Shoaib Khalid,† and Igor Kaganovich*,†

†*Princeton Plasma Physics Laboratory, Princeton, NJ 08540, USA*

‡*Department of Chemistry, Princeton University, Princeton, NJ 08544, USA*

E-mail: polyachenkoya@princeton.edu; ikaganov@pppl.gov

Sputtering yield at near-threshold energies

Here we provide a simple estimate of yield at projectile energies slightly above the sputtering lower energy threshold $E_{\text{sputt,S}}$. More precisely, we provide a lower bound on the yield, meaning we predict at least this much yield, though it is possibly higher if other sputtering pathways activate at higher energies. The main task is to connect the sputtering probability from the most susceptible hit-point $P_{\text{sputt}}(E_{\text{Ar}}|\text{optimal-hit})$ (Figure 2) to an experimentally measurable yield, which comes from uniform impacts of all hit-points. The key assumption we make for this estimate is that hit-points closer than in-plane thermal fluctuations of the top-layer atoms behave similarly. This allows us to estimate that if sputtering from the most-susceptible hit-point becomes possible at $E_{\text{Ar}} > E_{\text{sputt,S}}$, then sputtering from points within $\sim \langle \sigma_{xy} \rangle$ from the most susceptible point also becomes possible. For the order-of-magnitude estimate, we also assume the sputtering probability from these points $P_S(x, y) = P_{\text{sputt}}(E_{\text{Ar}}|\text{optimal-hit})$ does not depend on the hit-point within this small circle. For a given elementary triangle, the 1/6 fraction of the points within $\langle \sigma_{xy} \rangle$ lies inside the triangle. This gives the lower bound on the yield

$$Y_S(E_{\text{Ar}}) \geq P_{\text{sputt}}(E_{\text{Ar}}|\text{optimal-hit}) \frac{\pi \langle \sigma_{xy} \rangle^2 / 6}{S_{\text{triangle}}} \tag{S1}$$

Approximate equality is realized when no additional sputtering pathways, except for one found at $E_{\text{Ar}} = E_{\text{sputt,S}}$ activate at $E_{\text{Ar}} > E_{\text{sputt,S}}$. If such new pathways activate, yield can be much higher.

We aim at a lower bound for yield, so we use a conservative (low) $\langle \sigma_{xy} \rangle \sim 0.1$Å (Figure S9). We use $a_{\text{lattice}} = 3.15$Å (Figure S5) for $S_{\text{triangle}} = a_{\text{lattice}}^2 / 4\sqrt{3}$ and get $Y_S(E_{\text{Ar}}) > 0.0036 \cdot P_{\text{sputt}}(E_{\text{Ar}}|\text{optimal-hit})$. Given $|P_{\text{sputt}}| \sim 1$, the order of magnitude $Y_S \sim 10^{-2.5}$ is consistent with the yield reported near the lower sputtering energy threshold for MoS_2 in MD simulations.[1]

As addressed in Figure S7, precise quantification of yield near the sputtering threshold

depends on exact definitions of sputtering vs assisted desorption. Additionally, yield per impact depends on the degree of damage already present near the hit-point, which makes impacts history-dependent and forces simulating many consecutive events for the same material. Finally, systematic quantification of yield requires sampling of all hit-points in the elementary triangle (Figure 1d). Such calculations require orders of magnitude more sampling than reported here, which is why they are often conducted via force-field MD.[1]

The same logic also allows us to estimate how to compare our sampling N_{hits} per a fixed hit-point to the usual sampling per elementary triangle $N_{\text{sample}} \sim N_{\text{hits}} \frac{S_{\text{triangle}}}{\pi \langle \sigma_{xy} \rangle^2} \approx 270 \cdot N_{\text{hits}}$. Thus, given the minimal sampling $N_{\text{hits,min}} = 14$ used in this work, this translates into $N_{\text{sample,min}} \approx 4000$.

Surface coverage fraction effects

The sputtering through the formation of (meta)stable species such as SO_2 relies on a high surface-coverage assumption because forming such products requires having several functionalizing atoms near each other. Lower surface coverage will decrease the probability of the proposed sputtering mechanisms. The fluorine case is more complicated and most likely requires AIMD studies to quantify coverage fraction effects. The MoS_2O case, however, has a simpler, cleaner sputtering mechanism, which allows us to estimate the oxygen surface coverage fraction necessary to make the SO_2 sputtering mechanisms dominant. At a coverage fraction c, each SO pair has $N = 3$ neighboring sites that can host O atoms. The probability that n of them are occupied is $C_n^N c^n (1-c)^{N-n}$. Only reflections of the impacted O into a neighboring SO activates the proposed sputtering pathway, so the probability to activate it decreases by n/N compared to full coverage. Thus, the probability to result in sputtering given an impact at the most susceptible hit-point at a random thermal fluctuation is

$$P_{\text{sputt}}(\ldots, c < 1) \approx P_{\text{sputt}}(\ldots, c = 1)\cdot\sum_{n=1}^{N}\frac{n}{N}C_n^N c^n(1-c)^{N-n} = ... = P_{\text{sputt}}(\ldots, c = 1)\cdot c \quad \text{(S2)}$$

Interestingly, this result does not depend on N. Other summation weighting may result in a different coefficient in front of c, which may depend on N. But the expression always will be $\alpha c + \mathcal{O}(c^2)$ with $\alpha \sim 1$. This tells us that if we want the yield Y_S of the given sputtering pathways to decrease no more than by $\gamma = 0.1$ times due to decreased surface coverage, then from $P_{\text{sputt}}(\ldots, c < 1)/P_{\text{sputt}}(\ldots, c = 1) > \gamma = 0.1$ the surface coverage should be $c > \gamma = 0.1$.

Sputtering mechanism differences for MoS_2O vs MoS_2F

We hypothesize that forming SO_2 is the key step for MoS_2O sputtering (Figure 3), which will inevitably lead to thermal desorption of SO_2. This means that only a single S-O bond needs to be broken by Ar to enable such a sputtering mechanism. Forming SF_n products, on the other hand, may not be sufficient for their subsequent thermal desorption because they are much more reactive. Thus, weakening of the Mo-S bonds by F atoms (due to high electronegativity of fluorine) may be a necessary part of the decrease in the sputtering energy threshold.

A chemical reason for symmetry breaking in MoS_2F, but not in MoS_2O can be impacted by the electronegativity difference between F and O. The more electronegative F can pull more electron density from S. The S-F bond is also longer than S-O ($d_{bond} \approx 1.9$ Å vs 1.4 Å, see Figure S3a). This creates a stronger dipole $\sim d_{bond}q_{bond}$ in each S-F pair. Such dipole formation was reported for MoS_2O.[2] All dipoles are aligned, making them mutually repulsive, and this repulsion is stronger for S-F dipoles. Thus, this may impose sufficient stress on MoS_2 to break its symmetry.

Additionally, adding an F atom to the MoS_2 unit cell adds odd number of electrons, while

adding an O adds an even number of electrons. There is a well-known “Peierls instability” that was first explained in 1D,[3,4] but later “Peierls distortions” were found in 2D and 3D.[5,6] All these effects are driven by the fact that unit cells with odd number of electrons can undergo “spontaneous” symmetry breaking that splits the degeneracy in electron bands near the edge of the Brillouin zone and thus the valence band decreases its average energy. We believe that distortions of F atoms from positions directly above their host S atoms is a case of such “Peierls distortions”.

We note that typical sputtering events reported here occur on the timescale < 1 ps, as shown in Figures 3 and S1. This does not mean sputtering is impossible at lower energies; for example, even thermal sputtering on a timescale of months is feasible.[7] We also observed a multi-step S-vacancy creation process on timescales as long as ~ 20 ps (shown on Figure S7). However, such events exhibit a clear separation of timescales between the product formation and product removal. An Ar impact that is not sufficient for fast ejection of products can still catalyze product formation, with subsequent thermal desorption occurring on a much longer timescale. Since most of the immediate sputtering events we observed occurred within < 1 ps, we chose 2 ps as the timescale to separate ”immediate” sputtering from thermal desorption of products. Thus, we simulate for 2 ps after the impact and conclude that no sputtering has occurred if the products do not detach from the material within this time.

Angular sensitivity $\Leftrightarrow$ ground-state order

Fluorinated MoS_2, which is less ordered than MoS_2O even before the impact, shows no systematic variation with increasing impact angle. This indicates that once the symmetry is disrupted, further changes in the direction of incoming projectiles have little additional effect on the sputtering behavior. This is because the main effect of a slight tilt of the impact from the surface normal is to allow the impacted O/F to pass by the S atom directly below it, thereby enabling the O/F to break its bond with the host S atom and potentially

initiate the formation of sputtering products. Such tilting is necessary for MoS_2O where O atoms sit directly on top of S atoms, whereas F atoms are already slightly shifted from their underlying S atoms, so small additional tilts do not produce a significant change.

The in-plane angle φ is also shown to be important for MoS_2O (Figure S10). We found in-plane angles that maximize sputtering to be $\varphi_{\mathrm{opt}}(O) = 30°$ and $\varphi_{\mathrm{opt}}(S) = 0°$. Fluorine did not show sensitivity to φ. This is consistent with proposed sputtering mechanisms: Pristine MoS_2 is sputtered by physically breaking the bonds of a single S atom, which requires that other in-plane atoms do not obstruct the S atom as it gains maximum displacement, while still preventing it from penetrating too deeply into the material in order to minimize intermediate collisions. This condition is achieved at $\varphi = 0°$ (see Figure 1d) where the impacted S atom has the largest available in-plane space in the reflection direction. The hit-point at the center of the hexagon (which was found to be optimal for MoS_2) leads to the projectile energy being distributed approximately equally among three Mo atoms, such that no single Mo atom deviates too far from its equilibrium position and collides with its neighbors. The temporary storage and redistribution of projectile energy in this case is more complex and likely involves several many-body effects. The mechanism for MoS_2O, on the other hand, involves the formation of SO_2, which subsequently escapes. Impacting an O atom at $\varphi = 30°$ directs it toward its nearest S neighbor that has another O atom attached (see Figure 1d). Thus, SO_2 formation is expected to be maximized at $\varphi = 30°$, with the hit-point directly on the oxygen. We also quantify the φ dependence (Figure S10b) for a fixed $\theta = 10°$ bit enough to see benefit from tilting θ. Approximately 25% of the φ range ($\sim [25; 40]°$ out of $[0; 60]°$) shows optimal energy threshold values within errorbars. Threshold for all other φ values can be obtained by symmetry (Figure S10c). Blue dashed lines on Figure S10c show how 6 energy threshold minima match with the 6 directions at 6 neighboring oxygen atoms (given the hit-point is at an oxygen atom). This further confirms out interpretation. Finally, the insensitivity of MoS_2F to φ again highlights how structural disorder suppresses its directional response.

Finding the most damage-susceptible hit point

An analysis similar to Figure S4 is also performed for MoS_2F. It shows that the site above sulfur is among the most susceptible. Different F atoms are displaced by different amounts from being directly above their S atoms, which means that a head-on collision requires a hit-point slightly offset from the directly above-sulfur position. We speculate that the effect of this shift is negligible, because the directional insensitivity of MoS_2F arises not only from the random strong displacements of the equilibrium positions of the directly impacted F atoms, but also from the surrounding F atoms. These surrounding F atoms will still not be sufficiently ordered even if the initial Ar-F impact is head-on. Analogous tests for MoS_2 find the most susceptible point to be at the center of the 3Mo-3S hexagon (the Ar position in Figure S1 at t=0). We speculate that this is because it is the most effective hit-point for the sputtering mechanism shown in S1. This mechanism is likely the most energy-efficient, as the reversal of the projectile velocity occurs via "reflection" from the Mo layer through approximately equal displacement of three Mo atoms. As a result, the maximum Mo displacement is minimized, reducing Mo-Mo collisions with neighboring atoms and thereby minimizing energy losses.

Simple theory for MoS_2O $E_{\mathrm{sputt}}(T)$

The qualitative difference in the angular dependence $E_{\mathrm{sputt}}(\theta)$ between MoS_2O and MoS_2F has important implications for their temperature dependence since thermal fluctuations lead to a finite spread in reflection angles even for orthogonal impacts. This angular spread is non-negligible because the magnitude of thermal fluctuations of O and F atoms in the material plane is comparable to their hard-core repulsion interaction distance with Ar (see Figure S11). This means that even orthogonal impacts will not always result in strictly vertical momentum transfer. Instead, thermal fluctuations of O/F atoms make the collision non-head-on, thus introducing a distribution of reflection angles (see SI and Figure S11):

$$\theta_T(E_{\text{Ar}\perp}) \approx \arcsin\left(\frac{\sigma_{xy,X}(T)s_N}{rArX(E_{\text{Ar}\perp}, \theta_T(E_{\text{Ar}\perp}))}\right) \tag{S3}$$

where $\sigma_{xy,X}(T)$ represents the magnitude of thermal fluctuations of X={O,F} in the plane of MoS_2, (see Fig S9 for its temperature dependence). The scale factor $s_N = \chi_1.PPF(1 - 1/(N+1))$ comes from our definition of the sputtering threshold. We have a finite sample of N impact trials, and we register sputtering if at least one trial results in sputtering. This means that the maximum fluctuation of magnitude σ over all N trials has the probability of exceeding σ_N equal to the probability of a single fluctuation exceeding σ. Therefore, we use $\sigma_N = \sigma s_N$ as a characteristic fluctuation size instead of just σ. In a normal case, σ_{xy} would be distributed $\sim \chi_2 = \sqrt{\chi_2^2}$ due to $\sqrt{\sigma_x^2 + \sigma_y^2}$. However, only a narrow range of $\varphi_{opt} \pm \delta\varphi$ angles is susceptible to damage near the sputtering energy threshold, with $\delta\varphi \sim 8°$ (Figure S10b), so only deviations along a single axis $\varphi \sim \varphi_{opt}$ are relevant, and the distribution reduces to χ_1 in our case. Since we require the probability of observing a deviation larger than σ_N at least once in N trials to be significant, we set $\chi_1.CDF(\sigma_N/\sigma) = 1 - 1/(N+1)$, where $N+1$ instead of N is used to remove singularity at $N+1$. This means that a single fluctuation has a probability $1/(N+1)$ of exceeding σ_N. Thus $\sigma_N = \sigma \cdot \chi_1.CDF^{-1}(1 - 1/(N+1)) = \sigma \cdot \chi_1.PPF(1 - 1/(N+1))$. For large N, this quantity has a weak dependence of $\approx \sqrt{2\ln(N+1)}$, therefore the exact number of trials is not substantially important.

The term $rArX$ denotes the Ar-X interatomic distance, defined as the sum of hard sphere radii that approximate the interacting atoms during the collision. It is determined by how closely the head-on component of the collision energy, $E_{\text{Ar}\perp}\cos^2(\theta)$ brings the atoms together. Thus, it depends on the impact energy and collision geometry as described below (see SI for details):

$$U_{\text{Ar-X}}(r_{\text{Ar-X}}) = \frac{E_{\text{Ar}\perp}\cos^2\theta}{1 + m_{\text{X}}/m_{\text{Ar}}} \tag{S4}$$

where $U_{\text{Ar-X}}$ is the two-atom potential energy surface (PES) between Ar and X. We

take its inverse function $U^{-1}_{\text{Ar-X}}$ such that $r = U^{-1}_{\text{Ar-X}}(E)$ is the interatomic distance at which $U_{\text{Ar-X}}(r) = E$. The factor $1 + m_{\text{X}}/m_{\text{Ar}}$ comes from the center-of-mass frame treatment of collision.

For typical relevant $(E_{\text{Ar}\perp}, T)$ ranges, the in-plane thermal fluctuations are smaller than the collisional radii of the atoms, which results in a narrow spread of the reflection angle θ_T. More precisely, $\sigma_{xy,X}(T) \ll r_{\text{Ar-X}}(E_{\text{Ar}\perp}, \theta_T(E_{\text{Ar}\perp}))$, which leads to $\theta_T \ll 1$. Therefore, we can write $rArX(E_{\text{Ar}\perp}, \theta_T(E_{\text{Ar}\perp})) \approx r_{\text{Ar-X}}(E_{\text{Ar}\perp}, 0) = r_{\text{Ar-X}}(E_{\text{Ar}\perp})$. We can also assume a harmonic potential well for small $\sigma_{xy,X}(T)$, which leads to $\sigma(T) = \sigma_0\sqrt{T/T_0} \propto \sqrt{T}$ where $\sigma_0 = \sigma_{xy,X}(T_0)$. The scaling $\sim \sqrt{T}$ is confirmed in Figure S9. Combining this, we can write an approximate explicit expression

$$\theta_T(E_{\text{Ar}\perp}) \approx \arcsin\left(\frac{\sigma_0 s_N}{r_{\text{Ar-X}}(E_{\text{Ar}\perp})}\sqrt{\frac{T}{T_0}}\right) \approx \frac{\sigma_0 s_N}{r_{\text{Ar-X}}(E_{\text{Ar}\perp})}\sqrt{\frac{T}{T_0}} \tag{S5}$$

Thus, an O or F atom fluctuating at temperature T and impacted by an Ar atom with orthogonal energy $E_{\text{Ar}\perp}$ can acquire velocities distributed within an angular range of $\theta \approx 0 \pm \theta_T(E_{\text{Ar}\perp})$.

On the other hand, it is possible to isolate the effect of impact non-normality from thermal noise by bombarding surfaces equilibrated at temperatures low enough that thermal fluctuations are negligible, meaning $\sigma_{xy}(T) \ll d_{\text{atom,X}} = r_{\text{Ar-X}}(E_{\text{sputt,min}})$. The results are shown in Figure S8a. No significant angle dependence is observed for MoS_2F, as expected from its irregular ground state structure. However, MoS_2O exhibits a non-linear decrease in $E_{\text{sputt}}(\theta)$, dropping by $\sim 40-50\%$ at $\theta \sim 10-15°$ before flattening. Although not shown in the plot, it starts rising at some point θ_{opt} between 30° and 45°. The curve $E_{\text{Ar-X}}(\theta < \theta_{\text{opt}})$ therefore represents the energy threshold that Ar must have in a head-on, non-normal collision with X to induce ejection of an S atom.

Thus, eq.(S3) gives the range of reflection angles that are thermally accessible (purple shade on Figure S8b), while the inverted curve $E_{\text{Ar-X}}(\theta)$ from Figure 4a provides the minimum reflection angles $\theta_{\text{Ar-X}}(E_{\text{head-on}})$ required for sputtering (blue shade on Figure S8b). As the

angular spread increases with T, once angles $\theta > \theta_{\text{Ar-X}}(E_1)$ become thermally accessible at a certain temperature T_1, sputtering with $E_{\text{head-on}} = E_1$ becomes frequent at $T > T_1$. See the SI text for the derivation of the purple curve in Figure S8b. Solving eq.(S6) for X=Oxygen numerically gives the red dashed line in Figure 5a.

2-body collision model

To translate the head-on, non-normal energy threshold $E_{\text{Ar-X}}(\theta)$ into a threshold for a non-head-on but normal impact, $E_{\text{Ar}\perp}(\theta)$, we recall that only a factor of $\cos^2(\theta)$ of the total energy participates in the collision when the reflection angle is θ. This yields $E_{\text{Ar}\perp}(\theta)\cos^2(\theta) = E_{\text{Ar-X}}(\theta)$. This results in a system of two equations with two unknowns $(\theta_T, E_{\text{Ar}\perp})$:

$$\begin{cases} U_{\text{Ar-X}}\left(\dfrac{\sigma_{xy}(T)}{\sin(\theta_T)}\right) = \dfrac{E_{\text{Ar}\perp}\cos^2(\theta_T)}{1+m_{\text{X}}/m_{\text{Ar}}} \Rightarrow \theta_T(E_{\text{Ar}\perp}) \\ E_{\text{Ar}\perp} = \dfrac{E_{\text{Ar-X}}(\theta_T(E_{\text{Ar}\perp}))}{\cos^2(\theta_T(E_{\text{Ar}\perp}))} \Rightarrow E_{\text{Ar}\perp}(T) \end{cases} \tag{S6}$$

where the first equation describes the spread of reflection angles of the impacted O/F atoms and the second equation relates the normal, non-head-on impact energy (which is often controlled experimentally) to the non-normal head-on energy (which is easier to simulate and is temperature independent to a good approximation).

The two equations correspond to two physical conditions required for sputtering in the proposed mechanism: thermal fluctuations of the impacted O/F should be wide enough to allow the reflection angle spread θ_T that includes angles sufficient for sputtering according to $E_{\text{Ar-X}}(\theta)$. This breaks down into 2 conditions:

1. At a given temperature T, normal Ar impacts induce a spread of reflection angles $\theta < \theta_T$. The purple region in Figure S8b corresponds to such a region for $T = 300$K. There is also a slight dependence on $E_{\text{Ar}\perp}$, because higher $E_{\text{Ar}\perp}$ result in "smaller" repulsive sizes of atoms.

2. At each angle θ, there is a minimal energy $E_{\text{Ar-X}}(\theta)$ that an Ar must have in a head-on collision at angle θ to sputter an S atom. This corresponds to the blue region on S8b

Combining the two, the blue region in Figure S8b represents pairs (head-on energy; angle) = $(E_{\text{Ar-X}}, \theta)$ that would result in sputtering, while the purple region represents the accessible spread of reflection angles that are realized during an impact. Therefore, sputtering happens for those $(E_{\text{Ar-X}}, \theta)$ values over which the two regions overlap, meaning that thermal fluctuations make the reflection angle spread wide enough to reach angles θ that result in sputtering at a given energy.

Finally, the inherent θ-spread of the projectiles is incorporated by using $\sqrt{\theta_T(E_{\text{Ar}\perp})^2 + \theta_{\text{inherent}}^2}$ as the new θ_T. Such simple quadratic addition of fluctuations is used because of two reasons. First, the angles are small and therefore add approximately linearly. Additionally, the inherent and thermal θ fluctuations are statistically independent, so their variances add linearly.

Derivation details

Derivation of eq.(S4) - (S6): Referring to Figure S11, we first transform to the center-of-mass(COM) frame as shown below:

$$m_{\text{O}} v_{\text{CoM}} = m_{\text{Ar}}(v_{\text{Ar}} - v_{\text{CoM}}) \quad \Rightarrow \quad v_{\text{CoM}} = \frac{m_{\text{Ar}} v_{\text{Ar}}}{m_{\text{Ar}} + m_{\text{O}}} \tag{S7}$$

We then decompose the collision into a head-on collision along the line connecting the centers of Ar and O (thick dashed line in Figure S11) and the orthogonal direction, which does not experience any changes after the collision. Along the head-on direction, we can write energy conservation to find the effective hard-sphere distance between Ar and O at their closest approach $r_{\text{Ar-O}}$:

$$\frac{m_{\text{O}}(v_{\text{CoM}}\cos(\theta))^2}{2} + \frac{m_{\text{Ar}}((v_{\text{Ar}} - v_{\text{CoM}})\cos(\theta))^2}{2} = E_{\text{collision}} = U_{\text{Ar-O}}(r_{\text{Ar-O}}) \tag{S8}$$

Relativistic corrections are neglected because $v_{\mathrm{Ar}}/c \sim 10^{-4}$ at $E_{\mathrm{Ar}\perp} \sim 10$ eV. Thus, the incoming Ar energy is $E_{\mathrm{Ar}\perp} = m_{\mathrm{Ar}} v_{\mathrm{Ar}}^2/2$. Plugging everything in, we get

$$E_{\mathrm{collision}} = \frac{E_{\mathrm{Ar}\perp} \cos^2(\theta)}{1 + m_{\mathrm{O}}/m_{\mathrm{Ar}}} \tag{S9}$$

which is what appears in eq.(S4).

The derivation of eq.(S3) follows directly from Figure S11: The triangle on points (Ar position; O current position; O equilibrium position) has the angle θ and $\pi/2$, and sides $\sigma_{xy,O}$ and $r_{\text{Ar-O}}$, thus $\sin(\theta) = \sigma_{xy,O}/r_{\text{Ar-O}}$, which is equivalent to eq.(S3).

Numerical solution for $E_{\mathrm{Ar}\perp}(T)$

Equations (S6) can be rewritten in a less intuitive form, which nevertheless simplifies mathematical analysis

$$\begin{cases} U_{\text{Ar-X}}\left(\dfrac{\sigma_{xy,X}(T)}{\sin(\theta_0)}\right) = \dfrac{E_{\text{Ar-X}}(\theta_0)}{1 + m_{\mathrm{X}}/m_{\mathrm{Ar}}} \quad \Rightarrow \quad \theta_0(\sigma_{xy,X}(T)) \\ E_{\mathrm{Ar}\perp}(T) = E_{\mathrm{Ar}\perp}(\theta_0(\sigma_{xy,X}(T))) = \dfrac{E_{\text{Ar-X}}(\theta_0)}{\cos^2(\theta_0)} \end{cases} \tag{S10}$$

where X is the impacted atom type, which can be O/F/S, and θ_0 is different from θ_T above and is just a function of σ.

A sufficient condition for solution uniqueness is $\theta_0 < \theta_{\mathrm{opt}}$ where $\theta_{\mathrm{opt}}(X) = \mathtt{argmin}_\theta(E_{\text{Ar-X}}(\theta))$. Oxygen is shown to satisfy at least $\theta_{\mathrm{opt}}(O) > 30°$. This gives the maximum applicable temperature condition

$$T < T_{\max}(X) = \sigma_{xy,X}^{-1}\left[\frac{\sin(\theta_{\mathrm{opt}}(X))U_{\text{Ar-X}}^{-1}(E_{\text{Ar-X}}(\theta_{\mathrm{opt}}(X)))}{1 + m_{\mathrm{X}}/m_{\mathrm{Ar}}}\right] \tag{S11}$$

where $\sigma_{xy,X}^{-1}(\sigma)$ is the inverse function of $\sigma_{xy,X}(T)$ such that $\sigma_{xy,X}^{-1}(\sigma_{xy,X}(T)) = T$.

We only need to consider $U(r < r_{\mathrm{well}})$, where $r_{\mathrm{well}} = \mathtt{argmin}_r[U(r)]$, because this is the range that contains the hard-sphere distance $r_{\text{hard-sphere}}$ that approximates the collision. The range $r < r_{\mathrm{well}}$ still covers the full range or energy values $[0; \infty]$, so both $\sigma_{xy}(T)$ and

$U(r < r_{\rm well})$ are always invertible. This is shown by shading the $r > r_{\rm well}$ on Figure S11b.

For oxygen, we can estimate $T_{\rm max}(\theta_{\rm opt}(O)) > T_{\rm max}(30°) \approx 4900$ K, which is beyond MoS_2 melting point of $T_{\rm melt} \sim 2650$ K.[8] Therefore, the theory is formally valid for all physically relevant temperatures. In practice, the breakdown is expected to occur earlier, when the harmonic-well approximation $\sigma_{xy}(T) \sim \sqrt{T}$, used here to extrapolate $\sigma_{xy}(T)$ in figure S9, begins to fail. A numerical evaluation of $\sigma_{xy}(T)$, would allow the applicable temperature range to be extended close to $T_{\rm melt}$.

High and low T regimes of $E_{\rm sputt}(T)$

The linear regime for low T comes from the fact that $E_{\text{Ar-X}}(\theta)$ in Figure 4 is quadratic at the origin $\theta = 0$, and $\cos(\theta)$ is also quadratic at $\theta = 0$. Thus, for temperatures T such that $\theta_T \ll 1$ we can expand $E_{\rm Ar\perp}(T) \approx E_0(1 - \alpha\theta_T^2)$. Next, we recall from eq.(S5) that for low T, $\theta_T \approx \sigma_0 / r_{\text{Ar-X}}(E_{\rm Ar\perp})\sqrt{T/T_0}$. The dependence of $r_{\text{Ar-X}}(E_{\rm Ar\perp})$ turns out to be weak for $E_{\rm Ar\perp} \sim 10$eV, allowing us to approximate $r_{\text{Ar-X}}(E_{\rm Ar\perp}) \approx d_{\rm atom,X} = \text{const}$ as shown below:

$$E_{\rm Ar\perp}(T) \approx E_0 \left(1 - \alpha \left(\frac{\sigma_0}{d_{\rm atom,X}}\right)^2 \frac{T}{T_0}\right) = E_0 - bT \qquad \text{(S12)}$$

The plateau regime is more incidental. It occurs when the decrease in $E_{\text{Ar-X}}(\theta_T)$ is compensated by the decrease in $\cos^2(\theta_T)$. It is wide compared to the linear region because $\sigma_{xy}(T) \propto \sqrt{T}$ changes more slowly at higher T, and $E_{\text{Ar-X}}(\theta)$ likewise varies more slowly at higher θ. The transition from the linear regime happens at T_{switch} when $E''_{\text{Ar-X}}(\theta)$ deviates from its initial parabolic shape, meaning the curvature $E''_{\text{Ar-X}}(\theta_{T_{switch}})$ becomes significantly different from $E''_{\text{Ar-X}}(0)$.

The theoretical prediction in Figure 5a is not followed exactly within the error-bars, which can be due to several approximations. For very small θ, the two-body approximation may break down, since the impacted O/F atom is pushed almost directly into the S atom below it, making the process at least a three-body problem. Too wide angles $\theta \sim 1$ may start

to break the hard-sphere collision model because higher θ leads to longer periods before the main collision when the Ar already feels the material below, which may alter its course in a more complicated way.

Spin treatment

Treating spins breaks down into two separate questions that are distinguished by their timescales: the appropriate equilibrium initial states and the appropriate dynamics during and after the collision. First, we explain why singlet initial states were chosen, and then why we decided that fixed-spin singlet dynamics was the optimal choice between computational cost and accuracy for dynamics.

First, we note that we used CP2K[9–18] to obtain results presented in this work, and we are only aware of global spin fixation within this framework. This can lead to unpaired electrons localizing on parts of the system where they are not desired, such as the impacting argon atom. Impacts with metastable projectiles can happen in reality, but are beyond the scope of this work. Simulating Ar impacts in a fixed triplet state lowers the sputtering thresholds relative to singlet results. However, the ground states of 2H phases of MoS_2, MoS_2O and MoS_2F are singlets (meaning these materials are not ferromagnetic). Thus, initializing the system in a triplet state puts it in an excited state configuration. We estimate it to be $\sim$ 1.8 eV above the ground state for a 4x4 single-layer MoS_2 + Ar system.

Oxygen is known to exhibit non-trivial spin effects. For example, its molecular form O_2 is known to have a triplet ground state. Figure S3b red lines show PES for an O atom at varying distances from MoS_2. It is significantly different for singlet (solid) and triplet (dashed) spin states of the systems. All simulations reported in the main text were performed in a fixed-spin singlet state, which may pose a question about their validity, since potential oxygen dissociation from MoS_2O can play a significant role in the processes investigated in this work. However, we provide the following qualitative arguments for why simulating

impacts in a fixed singlet state represents an optimal choice between computational cost and accuracy:

1. The main ejection product for MoS_2O is SO_2, which has a singlet ground state. We simulated fixed-triplet Ar bombardment of MoS_2O and the dominant product was still SO_2. The sputtering threshold in fixed-triplet simulations decreased by (2 ± 0.5) eV. This is consistent with our observation that the total system energy in the triplet state is 1.8 eV higher than in the singlet state, corresponding to an "unpairing excitation" of 1.8 eV.

2. Oxygen singlet is a ground state when attached to MoS_2 (Figure S3b) and it follows the singlet PES at least until $r_{S-O} \approx 2.15$ Å. Triplet becomes lower at larger r, which might be relevant for atomic O sputtering, for example during oxygen cleaning from the TMD after processing. However, we do not focus on O sputtering in this work.

3. While the triplet becomes lower than singlet for O for $r > 2.15$ Å, we believe that fix-spin dynamics is appropriate even for the impacts simulated here, because of the spin-relaxation dynamics of oxygen:

   (a) While estimating de-excitation time of a specific atomic O near a TMD has not been studied previously, we can try to get insight from molecular oxygen. Given a singlet state, O_2 requires $\approx 20\mu s$ to de-excite to a triplet state.[19,20] This is about ~ 7 orders of magnitude longer than the "immediate" sputtering processes (excluding long-term thermal desorption of products) considered in this work.

   (b) The $20\mu s$ estimate is under near-ambient conditions relative to ~ 10 eV collisions, which is a significant difference because de-excitations can only happen via relatively strong interactions coupling spin degrees of freedom to the rest of the system. Strong collisions can provide such coupling. Estimating de-excitation probabilities from collisions can be done in principle,[21] however, we did not perform such in-depth analysis because:

(c) Robust de-excitation to a triplet state can only occur at nuclei positions where the triplet electronic state is the ground state. This is not the case for O atoms attached to MoS_2 and remains true until $r_{S-O} = 2.15$ Å (Figure S3b). Therefore, the main Ar-O collision happens while O is in a position where its ground state is a singlet, thus it is not converted into a triplet.

(d) By the time the impacted O has moved more than 2.15 Å away from the top S-layer, the impact energy has likely dissipated into many surrounding atoms, and no single collision is likely to be sufficiently energetic to provide strong spin-electronic coupling capable of inducing a spin-flip.

DFT applicability

The Born–Oppenheimer (BO) approximation assumes that electrons remain in their ground state for each nuclear configuration, which requires that nuclei move much more slowly than electrons. An order of magnitude estimate for the lower boundary of electron velocity in atoms is the atomic unit $v_e = \alpha c$, where c is the speed of light and $\alpha = e^2/\hbar c \approx 1/137$. An Ar atom moving at $v = v_e$ has a kinetic energy of ≈ 1 MeV, which is far greater than 10 eV. All other energies in the collision are smaller than this, so the BO approximation should hold well during the whole process.

The ground-state approximation is more complicated. A necessary condition for it is the prevalence of ionic stopping power relative to the electronic one. It was experimentally shown for ice bombardment with He+ ions that ion energies below $\sim 1-2$ keV are dominated by ionic scattering.[22] One reason for this is the highly non-unitary mass ratio between electrons and ions, which suppresses fast energy transfer between ionic and electronic degrees of freedom.

However, excitations can be transferred directly into the electronic degrees of freedom of the material by several channels, such as impacts of excited atoms (Ar*) and/or neutraliza-

tion of the projectile shortly before impact. The latter can also be viewed as a special case of de-excitation, since a charged particle (ion) near a material with much higher capacitance is in an excited state. De-excitations can occur through a number of Auger and resonant like channels.[23] The resonant channel may not result in excitations because the electron transitions between states at the same energy level. It has been shown to dominate for Ne+ neutralization during MoS_2 bombardment at $E \leq 2$ keV due to a match between the electronic band structure of MoS_2 and the electronic levels of Ne+.[24] This is not always the case, for example $\sim 1/5$ He+ ions caused electron ejections from ice at $E_{ion} = 10$ eV[25] due to Auger neutralization. In this work, we do not account for excitations, which may affect quantitative results such as the absolute value of E_{sputt}. However, the qualitative relative results, such as different sputtering mechanisms and the decrease in E_{sputt} they induce do rely on simple physical explanations rather than DFT results alone, and we therefore expect them to hold in reality.

DFT and AIMD details

We used UZH pseudo-potentials GTH-GGA[26] and the corresponding basis sets TZV2P-MOLOPT-GGA-GTH,[13] as they are an update to the default CP2K GTH choice optimized for heavier elements such as transition metals and for mGGA functionals like R2SCAN, which we employed in several parts of this work. The Gaussian plane wave (GPW) method was used for propagation.[26–30] The CUTOFF and REL-CUTOFF parameters were converged to 400 Ry and 40 Ry, respectively, corresponding to an energy error tolerance of $\sim 10^{-2}$ H following the official CP2K guidelines. Achieving a tolerance of $\sim 10^{-3}$ H would require 700 Ry and 60 Ry respectively. Given the energy scale of our processes ~ 10 eV with $\Delta E/E \sim 0.1$, we selected the coarser $\sim 10^{-2}$ H accuracy. A unit cell of 4x4 was used for main results. No k-point sampling was done, meaning a 1x1x1 grid and always the Γ point. Oxygen adsorption energies computed at these parameters compared to a 5x5 unit cell with a 6x6x1

k-point grid differ by only ~ 0.3eV, which we consider as acceptable given the energy scale of ~ 10 eV of energies under study. ASPC extrapolation was used to predict the next step density.[31,32] Quickstep EPS was set to 10^{-8}, SCF EPS to 10^{-5} and orbital transformation (OT) diagonalization[33] was used with a FULL-SINGLE-INVERSE preconditioner and DIIS optimizer.[34] All considered TMDs are not metallic and/or magnetic, which allowed us to avoid smearing. A potential interaction cutoff of 10 Å was applied, and a time integration step of 1 fs was used. Impact simulations were performed in NVE ensemble to accurately capture energy transfer between system components and dynamics. Each simulation was run for 2 ps after the impact, as justified in the main text. Equilibration was performed in the $NP_{xy}L_zT$ ensemble and propagated until lattice vectors' convergence. The initial Z-distance of Ar atom from the top TMD layer was set to 10 Å.

The timestep of 1 fs is sufficiently small to ensure dynamically accurate trajectories of length ≤ 2 ps considered in this work. Dynamical accuracy here means that trajectories of all atoms in the system do not deviate significantly from exact solution of the dynamical ODE despite the errors from the finite timestep. In principle, such errors accumulate and lead to exponential divergence of MD trajectories from physical trajectories[35] even in the NVE ensemble, which was used for impact simulations. At long simulation times, such divergence leads to effectively sampling the NVE ensemble instead of producing a solution to the original dynamical ODE. However, there is a "dynamical memory time" t_m[35] before which such deviations are small and near-true dynamical trajectories are resolved. We want our collision events to be fully physical in the dynamical sense, not just in the NVE-sampling sense, because the proposed sputtering mechanisms can depend on e.g. synchronization of oscillations of several atoms, as it happens for forward-sputtering of S from pristine MoS_2. Therefore, we want the t_m to be larger than at least a typical active collision stage, which was found to take ~ 0.2 fs. The dynamical time was estimated for the LJ system to be $\sim 4\tau_{LJ}$, with log-dependence on the integration timestep. Making a rough approximation $\epsilon \sim 1$ eV, $\sigma \sim 1$ Å and $m \sim 20$ a.u., we get $\tau_{LJ} \sim 1.4$ ps. It gives us $dt \sim 10^{-3}\tau_{LJ}$,

thus $t_m(dt) \sim 5$ ps.[35] Therefore, the 0.2 ps of the active collision, and even the whole 2 ps collision events fit into the t_m. Thus, we can treat the obtained AIMD trajectories as true dynamical trajectories (as opposed to just NVE sampling) and use them to suggest time-resolved sputtering mechanisms.

Figure S3a shows only a negligible difference between the PBE[36] and R2SCAN[37] functionals for the PES, both in terms of adsorption energies and overall profiles. Dispersion corrections using rVV10[38] versus D3BJ[39] also show negligible differences. Therefore, we use the simplest option, PBE-D3. This choice is also commonly adopted in the literature for MoS_2 + O systems.[7,40,41] The R2SCAN functional combined with rVV10 dispersion was included for comparison because more sensitive properties, such as phonon spectra of MoS_2 has been shown to require R2SCAN + rVV10 for accurate description.[42] Our claim of a low adsorption energy barrier for atomic O and F is somewhat affected by the functional: PBE predicts a barrier closer to 0.1 eV, while R2SCAN yields a value closer to 0.2 eV. However, both values are comparable to ambient thermal energy $k_BT \sim 0.026$ eV. Therefore, the claim of unimpeded adsorption is likely justified.

Free-energy simulation setup

We used CP2K + PLUMED (the open-source, community-developed PLUMED library,[43] version 2.x[44] or alternatively version 1.x.[45]) to run meta-dynamics (metaD).[46,47] A 2x2 supercell with a single adsorbed O or F atom was employed. The distance between the adsorbing O/F atom $Z_{O/F}$ and the center of mass of the entire TMD layer Z_{TMD}, was chosen as the collective variable ($CV = \delta Z = Z_{TMD} - Z_{O/F}$) for metaD. We bias the Z-coordinate of the TMD layer to a fixed value with a parabolic potential $k_{TMD}(Z_{TMD} - Z_0)^2/2$ with $k_{TMD} = 2.5$ eV/Å^2. From unconstrained equilibration runs, we find that the stable bonding sites for both O and F are approximately on top of the corresponding S atoms. Therefore, we applied another parabolic bias ($k = 5$ eV/Å^2) on $X_{O/F} - X_{S,bond}$ and $Y_{O/F} - Y_{S,bond}$ to

prevent the O/F from slipping off the bonding S atom under the pressure of the metaD bias. Finally, we applied one-sided parabolic walls on δZ at 1.5 Å ($k = 3$ eV/Å^2) and 8 Å ($k = 1$ eV/Å^2) to prevent pushing the formed S-O/F bond into the layer and to avoid exploration of non-interacting regions and artifacts due to periodic boundary conditions. Gaussians of width 0.1 Å and height of 0.03 eV ($\sim k_B T$ to avoid trapping the CV) are deposited every 100 fs (which allows a few thermal motions of O/F in its potential well). A bias-factor of $\gamma = 100$ was chosen $\approx E_{desorb}/k_B T$ assuming a desorption barrier $E_{desorb} \sim 2 - 4$ eV and $T = 300$ K. Convergence analysis was performed according to the official guidelines (see Figures S3c and S3f). Convergence times, shown in the titles of Figures S3d and S3e, are $\sim 20 - 30$ ps. Each metaD calculation was run for at least 100 ps.

Figures

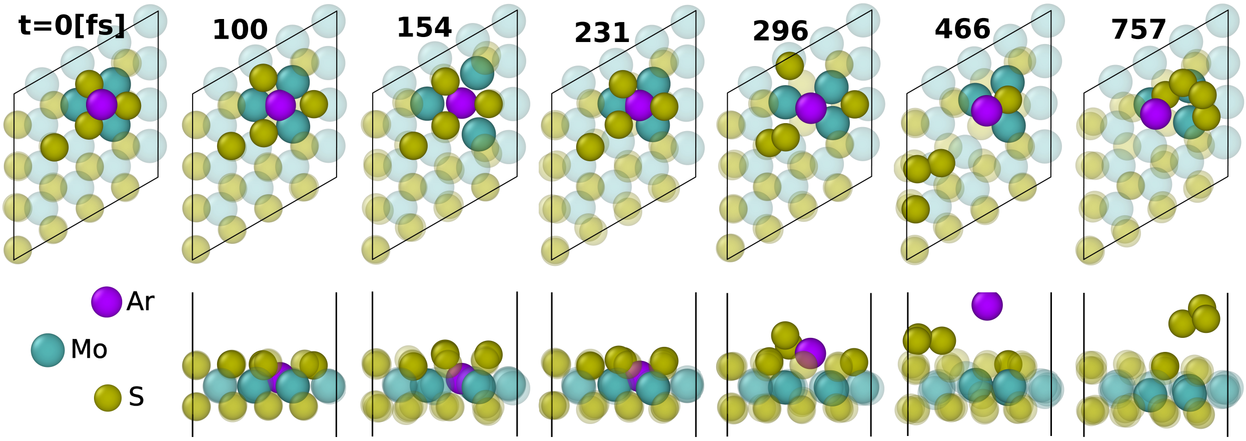

Figure S1: Typical sputtering of pristine MoS_2 at $E_{\mathrm{Ar}\perp} = 34$ eV. Video is given in video V2. (Top) plane view, (Bottom) side view. Gray numbers indicate timestamps in [fs]. The Ar atom has enough energy to penetrate through the top S layer and push apart Mo atoms (154 fs). These Mo atoms later push the Ar atom back out of the lattice as follows: First (231 fs), Ar remains located “within” the Mo layer, but the Mo atoms return from their displaced state. Then, the rebounding Molybdenum atoms strongly drive the Ar atom forward, which then collides with the top-layer S atoms at 296 fs. The displaced S-s subsequently combine to form an S_3 species that then depart. Using a search similar to one described in Figure S4, we showed that this mechanism is the most energy efficient.

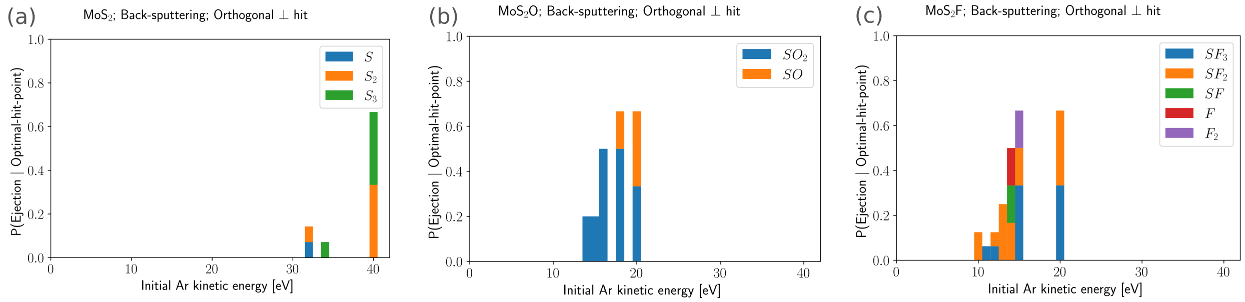

Figure S2: Breakdown of sputtering probabilities from Figure 2 by products. This further illustrates a cleaner sputtering nature for MoS_2O compared to more noisy sputtering observed for MoS_2F.

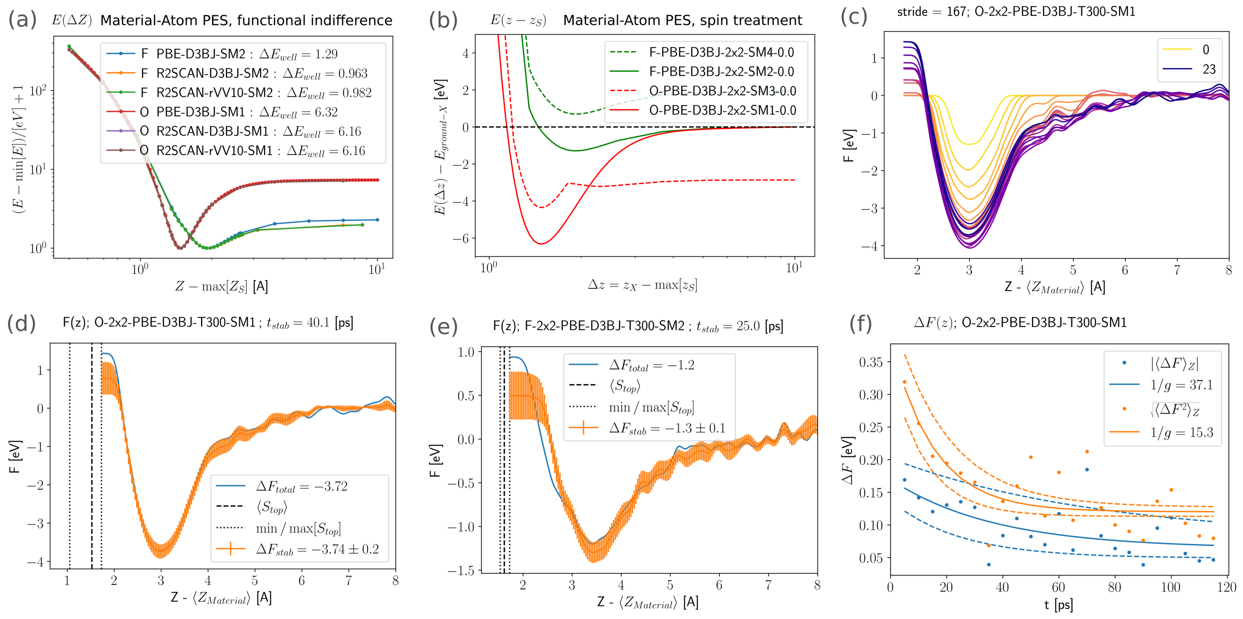

Figure S3: Calculations for O and F adsorption on MoS_2. (a) Potential energy profiles for placing an Oxygen (red) or Fluorine (green) atom at different distances from the top sulfur layer of a geometry-optimized MoS_2 2x2 single layer. The close agreement between R2SCAN and PBE curves indicates that using a mGGA is not essential. Similarly, the R2SCAN-rVV10 and R2SCAN-D3BJ curves suggest no significant advantage of rVV10 over D3BJ dispersion correction. (b) Spin-states analysis in a setup similar to A. Oxygen is red, Fluorine is green. Abrupt changes at small r are because we focused on plotting near-the-well points. Solid lines are what is used in AIMD collision simulations. Spin-excited (quintuplet) F-case is higher than doublet everywhere. The O-case triplet becomes the ground state at $r > 2.15$ Å. However, O is clearly a singlet when adsorbed, and there are no metastable states near $r = 2.15$ Å, which suggests O will be near $r = 2.15$ Å only for short times, making the spin-flips unlikely. The triplet PES is useful to confirm the previously reported dissociation barrier of ~ 1.3 eV. (c) Free-energy profile of an oxygen atom as a function of its distances from the center-of-mass of a 2x2 MoS_2 single layer. Dynamics are run with unconstrained spin to test whether dynamical spin polarization affects the profile near the singlet-triplet degeneracy region. (d) Same as (c), but for a fluorine atom. (e) A typical time-evolution of meta-dynamics free-energy profile. (f) A typical convergence analysis for meta-dynamics.

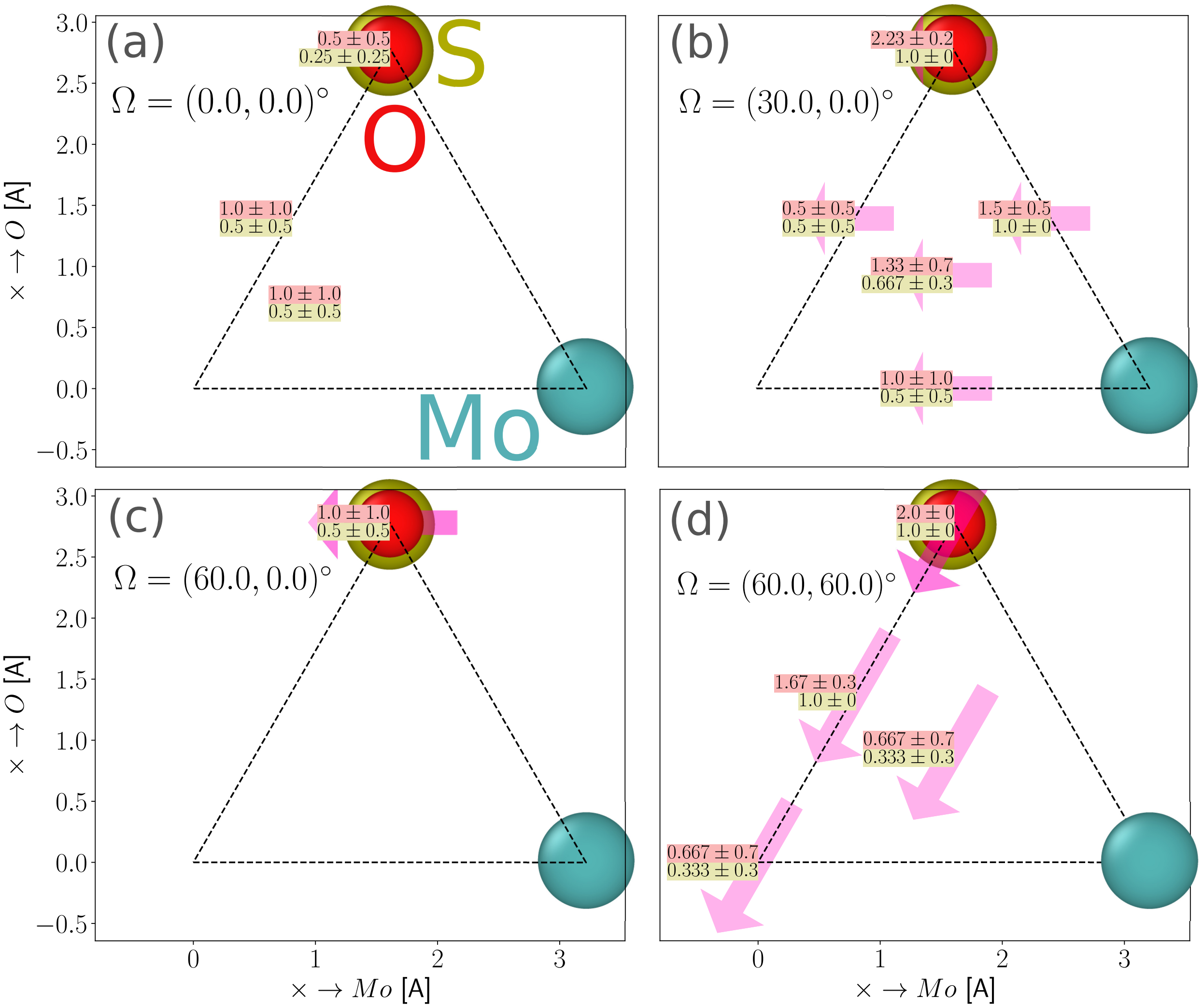

Figure S4: Finding the most damage-susceptible point of MoS_2O. Impacts are performed at $E_{\mathrm{Ar}} = 15$ eV. The triangle on each scheme corresponds to the purple triangle in Figure 1d, oriented according to the S and Mo atom labels. Thick purple arrows indicate projections of the initial Ar velocity. Different hit-points and impact angles are surveyed (a-d). The only hit-point that always results in sulfur sputtering is directly above sulfur, corresponding to a head-on impact on oxygen in its equilibrium position.

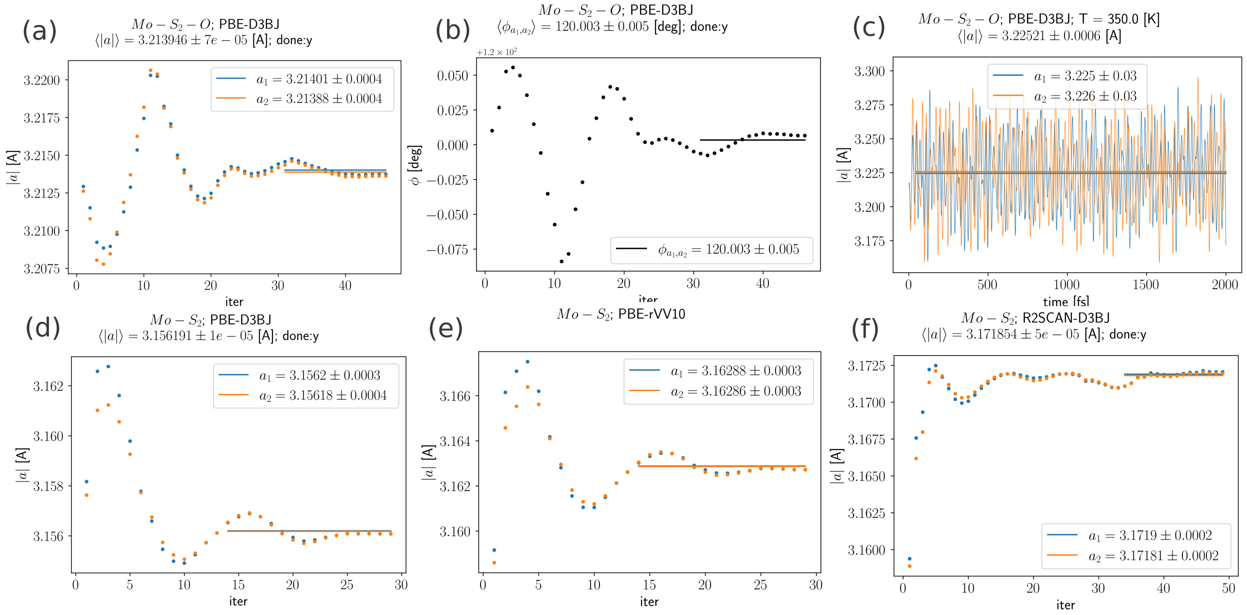

Figure S5: Equilibration before impact illustrated for MoS_2O. Geometry optimization was performed in the $NP_{xy}L_zT$ ensemble to allow the unit cell to break three-fold symmetry while preventing the collapse along the Z direction. (a) Evolution of both lattice constants during optimization. (b) Evolution of the angle between the lattice constant vectors. (c) $NP_{xy}L_zT$ AIMD trajectory confirming that the ground-state optimization result from (a) is close to the lowest free-energy state. (d) Geometry optimization of MoS_2. (e) Same as (d), but including rVV10 dispersion with parameters for TMDs.[42,48] The results in fact deviate even more from the experimental value of ~ 3.15 Å.[49] (f) Same as (d), but using R2SCAN functional with D3 dispersion parameters optimized for TMDs.[42]

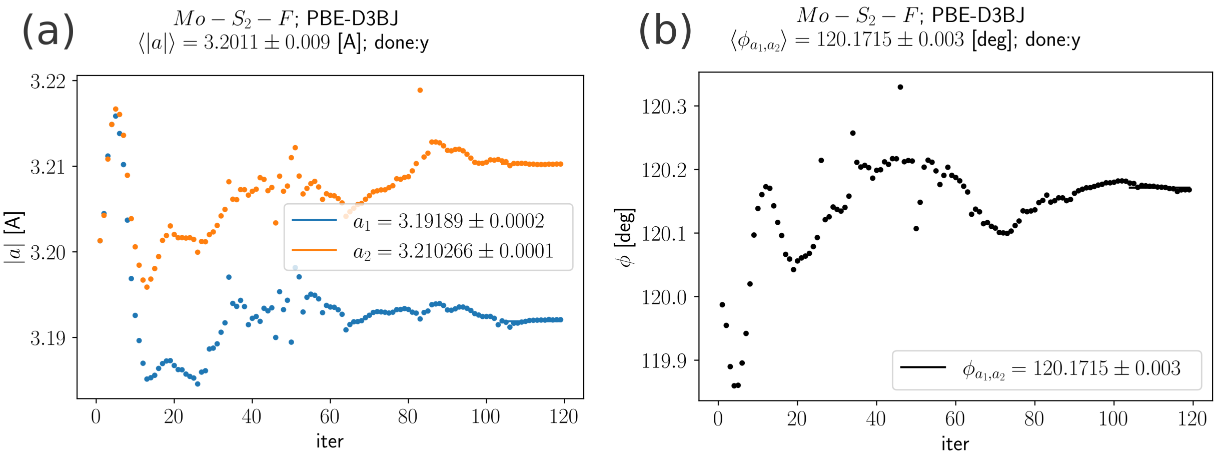

Figure S6: Geometry optimization of MoS_2F. PBE+D3 is chosen based on its comparison to alternatives in Figure S5. (a) Lattice constants evolution. Spontaneous symmetry breaking is observed, as the lattice constants differ well beyond the error bars. (b) Angle between lattice constant vectors, which also deviates significantly from 120° beyond the error bars.

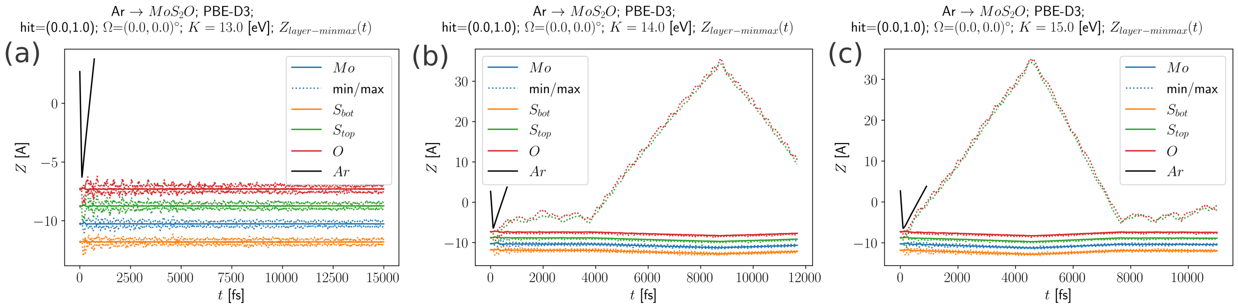

Figure S7: Sample longer AIMD runs verifying that a 2ps simulation time used for most production results is sufficient to identify immediate sputtering events. Red, Green, Blue, and Yellow correspond to the O, S_{top}, Mo, and S_{bot} layers, respectively. Dotted lines indicate the maximum and minimum Z-coordinates within each layer. An SO_2 forms on (b) at 14 eV, but takes several ps to desorb, while an impact energy of 15 eV is sufficient for immediate sputtering. In general, removal of sputtered products involves an activation process that can occur on a much longer timescale. However, such products can also form through mechanisms other than Ar impacts and would require a different analysis, which is beyond the scope of this work.

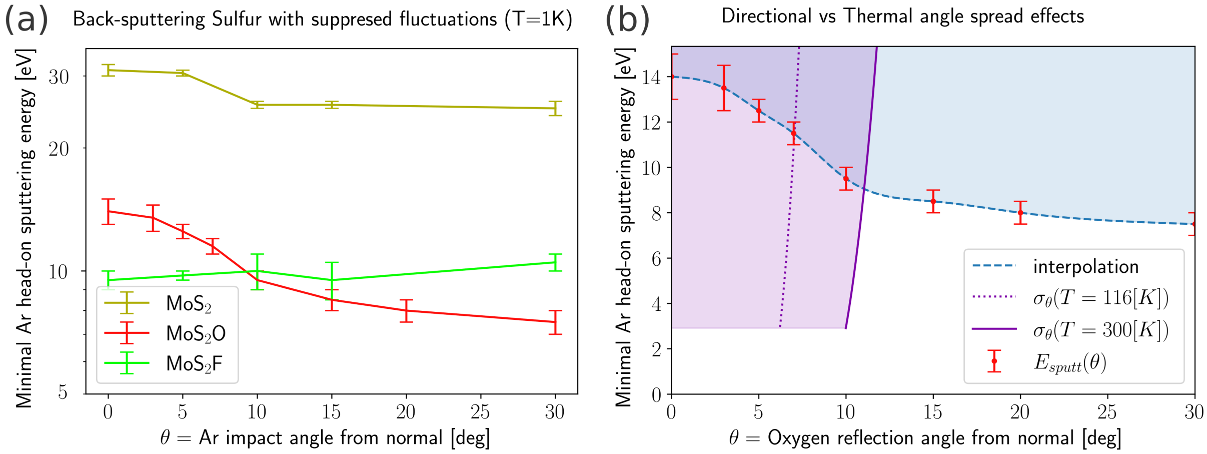

Figure S8: (a) Angular dependence of the sputtering threshold energy E_{sputt} for pristine (dark yellow), fluorinated (light green) and oxygenated (red) MoS_2 under normal hits. Such fluctuations are suppressed by setting $T = 1$ K. Each curve is obtained after optimizing the in-plane angle φ to minimize E_{sputt}, as shown for MoS_2O in Figure S10. Simulations at $\theta = 45°$ for MoS_2 and MoS_2O yielded $E_{MoS_2}(45°) > 35$ eV and $E_{MoS_2O}(45°) > 14$ eV. (b) Sputtering possibility diagram for MoS_2O. Red datapoints indicate the Ar energy threshold for sulfur ejections in non-normal head-on Ar-O collisions with suppressed thermal fluctuations (T=1 K). The blue dashed line shows a smooth (cubic) interpolation of the red data, and the blue shaded region marks where sputtering occurs. The solid purple line and corresponding shade represent the range of reflection angles θ that an O atom is likely to acquire after a Ar normal impact at $T = 300$ K, while the purple-dotted line shows the thermal spread θ_T accessible at $T = 116$ K. Overlap between the blue and purple regions indicates where sputtering from normal impacts is expected.

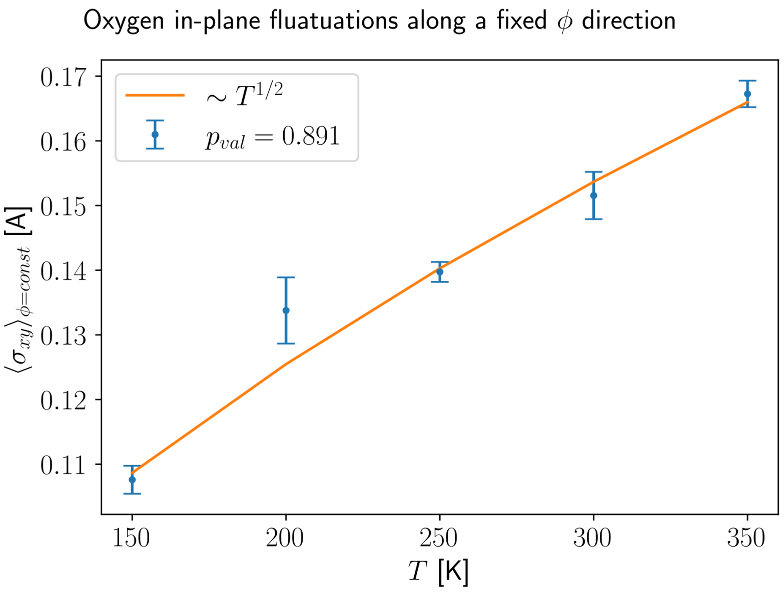

Figure S9: Magnitude of in-plane thermal fluctuations of oxygen atoms adsorbed on MoS_2. The $\sim T^{1/2}$ scaling follows from approximating the adsorbed atoms as confined in an approximately harmonic square well potential, which implies $\sigma_{xy}^2 \sim \langle U \rangle = \langle K \rangle \sim k_B T$ by the virial theorem. The high p-value indicates that the $T^{1/2}$ scaling provides a good description of the data. We redefine $\sigma^2_{xy,\varphi=\mathrm{const}} = (\sigma_x^2 + \sigma_y^2)/2$, where the factor 1/2 reflects that we are only interested in fluctuations along a relatively narrow range of in-plane angles $\varphi \approx \varphi_{\mathrm{opt}} \pm \delta\varphi$ with $\delta\varphi \sim 8°$ (Figure S10b). Only this range is susceptible to damage near the sputtering threshold E_{sputt}. We also assumed that in-plane fluctuations have the same magnitude in any in-plane direction, which is probably not exactly accurate because of in-plane inhomogeneity. However, σ_x was found to be within error-bars of σ_y in repeated simulations, which justifies computing a simple average for $\sigma^2_{xy,\varphi=\mathrm{const}}$.

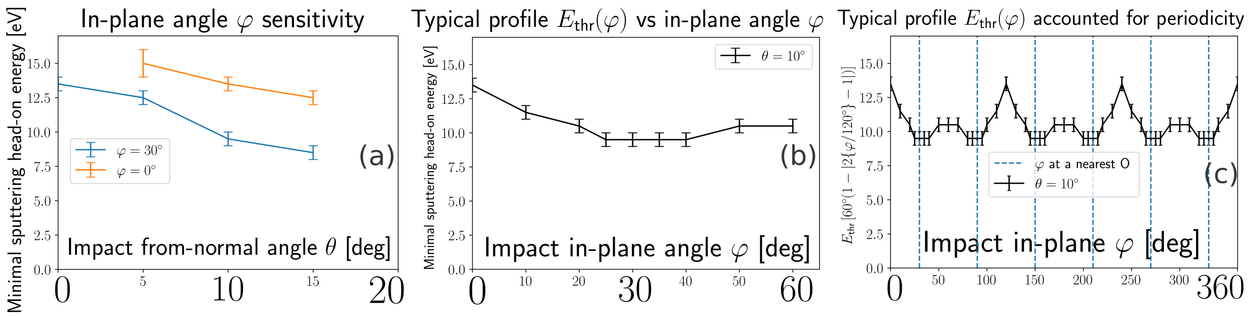

Figure S10: Importance of the in-plane impact angle φ for MoS_2O. (a) Clearly higher energies are required for sputtering at $\varphi = 0°$ than at 30°. We note that the curves do not have to be monotonic, at least for all $\varphi \neq \varphi_{\rm opt}$, since pushing an O more into the wrong direction φ can further complicate the sputtering process. We also note that the curves do not have to meet at $\theta = 0$ if fluctuations are suppressed because even an infinitesimal tilt in the wrong direction may complicate sputtering. In the presence of thermal fluctuations, the curves should meet for angles smaller than the thermal angular spread $\theta < \theta_T(E_{\rm Ar\perp})$. (b) Finer resolution for φ dependence at a fixed $\theta = 10°$. The θ is chosen big enough to make the θ-dependence clearly noticeable, but not too big so the obtained dependence may be interesting for thermally induced θ spread. The minima for $\varphi \in [25; 40]°$ corresponds to pushing the impacted O atom near-directly at a neighboring O atom, which is located at $\varphi = 30°$. (c) The data from (b), unwrapped using the symmetry (see Figure 1). The applied symmetry is only relevant for the elementary triangle corners (the purple triangle on Figure 1). The system is less symmetric for hit-points on triangle edges and even less so inside the triangle. Blue dashed lines note the direction at all 6 neighboring O-s.

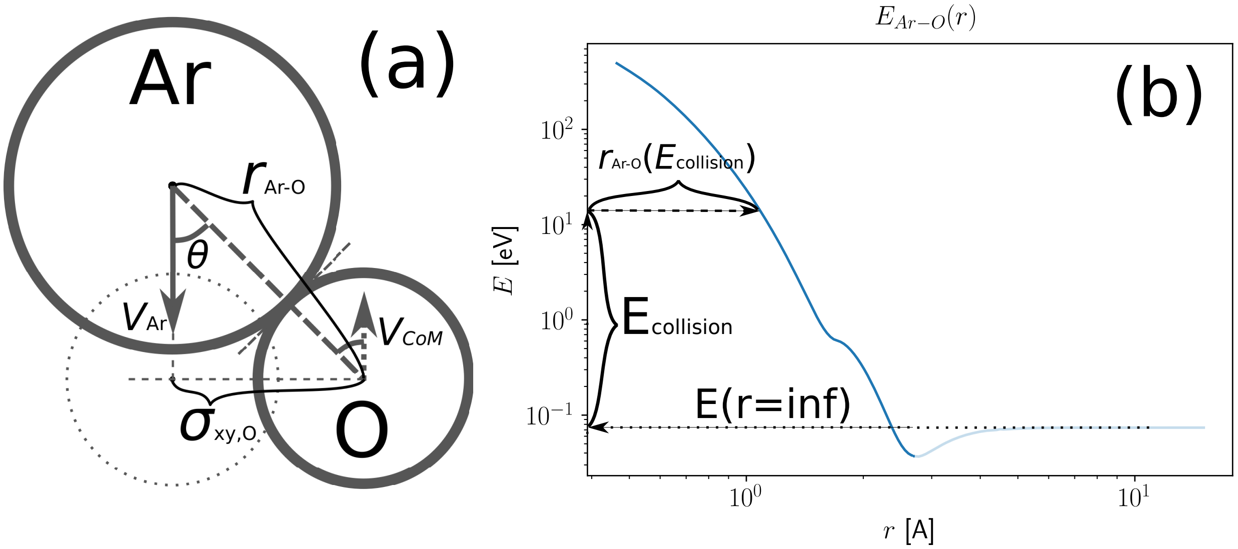

Figure S11: Ar-O collision schematic. (a) Hard-sphere Ar-O collision model. The dotted circle is the equilibrium O position, while the O circle shifted by $\sigma_{xy,O}$ to the right represents its position at the moment of impact with Ar. The collision plane (thin dashed line) is orthogonal to the line through the centers of Ar and O (thick dashed). The incoming Ar has velocity v_{Ar}, however treating the collision in the CoM frame assigns the O atoms a velocity v_{CoM} and subtracts the same v_{CoM} from v_{Ar}. Because $\sigma_{xy,O} \sim r_{\text{Ar-O}}$ we get non-negligible $\theta = \texttt{arcsin}(\sigma_{xy,O}/r_{\text{Ar-O}})$. A non-head-on collision can always be decomposed into a head-on collision and a fly-by (orthogonal to the head-on collision). (b) Two-atom potential energy profile $U_{\text{Ar-O}}(r)$ for Ar-O used in eq.(S4). The value of $r_{\text{Ar-O}}$ is found as $U^{-1}_{\text{Ar-O}}(E_{\text{collision}})$. See the Supplementary information for the derivation of $E_{\text{collision}}$.

References

(1) Kretschmer, S.; Maslov, M.; Ghaderzadeh, S.; Ghorbani-Asl, M.; Hlawacek, G.; Krasheninnikov, A. V. Supported Two-Dimensional Materials under Ion Irradiation: The Substrate Governs Defect Production. *ACS Applied Materials' Interfaces* **2018**, *10*, 30827–30836.

(2) Yan, H.; Chen, H.; Cui, X.; Guan, Q.; Wang, B.; Cai, Y. Unraveling energetics and states of adsorbing oxygen species with MoS2 for modulated work function. *Nanoscale Horizons* **2025**, *10*, 359–368.

(3) Kagoshima, S. Peierls Phase Transition. *Japanese Journal of Applied Physics* **1981**, *20*, 1617.

(4) Peierls, R. E. *Quantum Theory of Solids*; Oxford University Press, 2001.

(5) Burdett, J. K.; Lee, S. Peierls distortions in two and three dimensions and the structures of AB solids. *Journal of the American Chemical Society* **1983**, *105*, 1079–1083.

(6) You, H.; Zhang, Y.; Chen, J.; Ding, N.; An, M.; Miao, L.; Dong, S. Peierls transition driven ferroelasticity in the two-dimensional d-f hybrid magnets. *Physical Review B* **2021**, *103*.

(7) Pető, J.; Ollár, T.; Vancsó, P.; Popov, Z. I.; Magda, G. Z.; Dobrik, G.; Hwang, C.; Sorokin, P. B.; Tapasztó, L. Spontaneous doping of the basal plane of MoS2 single layers through oxygen substitution under ambient conditions. *Nature Chemistry* **2018**, *10*, 1246–1251.

(8) O'Neil, M. J. *The Merck Index — An Encyclopedia of Chemicals, Drugs, and Biologicals (Merck Index)*; Merck, 2006.

(9) Kühne, T. D.; Iannuzzi, M.; Del Ben, M.; Rybkin, V. V.; Seewald, P.; Stein, F.; Laino, T.; Khaliullin, R. Z.; Schütt, O.; Schiffmann, F. et al. CP2K: An electronic

structure and molecular dynamics software package - Quickstep: Efficient and accurate electronic structure calculations. *The Journal of Chemical Physics* **2020**, *152*.

(10) Hartwigsen, C.; Goedecker, S.; Hutter, J. Relativistic separable dual-space Gaussian pseudopotentials from H to Rn. *Physical Review B* **1998**, *58*, 3641–3662.

(11) Frigo, M.; Johnson, S. The Design and Implementation of FFTW3. *Proceedings of the IEEE* **2005**, *93*, 216–231.

(12) Krack, M. Pseudopotentials for H to Kr optimized for gradient-corrected exchange-correlation functionals. *Theoretical Chemistry Accounts* **2005**, *114*, 145–152.

(13) VandeVondele, J.; Hutter, J. Gaussian basis sets for accurate calculations on molecular systems in gas and condensed phases. *The Journal of Chemical Physics* **2007**, *127*.

(14) Grimme, S.; Ehrlich, S.; Goerigk, L. Effect of the damping function in dispersion corrected density functional theory. *Journal of Computational Chemistry* **2011**, *32*, 1456–1465.

(15) Borštnik, U.; VandeVondele, J.; Weber, V.; Hutter, J. Sparse matrix multiplication: The distributed block-compressed sparse row library. *Parallel Computing* **2014**, *40*, 47–58.

(16) Heinecke, A.; Henry, G.; Hutchinson, M.; Pabst, H. LIBXSMM: Accelerating Small Matrix Multiplications by Runtime Code Generation. **2016**, 981–991.

(17) Schütt, O.; Messmer, P.; Hutter, J.; VandeVondele, J. GPU-Accelerated Sparse Matrix–Matrix Multiplication for Linear Scaling Density Functional Theory. *Electronic Structure Calculations on Graphics Processing Units* **2016**, 173–190.

(18) Goerigk, L.; Hansen, A.; Bauer, C.; Ehrlich, S.; Najibi, A.; Grimme, S. A look at the density functional theory zoo with the advanced GMTKN55 database for general

main group thermochemistry, kinetics and noncovalent interactions. *Physical Chemistry Chemical Physics* **2017**, *19*, 32184–32215.

(19) Farmilo, A.; Wilkinson, F. On the mechanism of quenching of singlet oxygen in solution. *Photochemistry and Photobiology* **1973**, *18*, 447–450.

(20) Petrov, E. G.; Robert, B.; Lin, S. H.; Valkunas, L. Theory of Triplet Excitation Transfer in the Donor-Oxygen-Acceptor System: Application to Cytochrome b 6 f. *Biophysical Journal* **2015**, *109*, 1735–1745.

(21) Dagdigian, P. J.; Alexander, M. H.; Kłos, J. Theoretical investigation of the dynamics of O(1D→3P) electronic quenching by collision with Xe. *The Journal of Chemical Physics* **2015**, *143*.

(22) Famá, M.; Shi, J.; Baragiola, R. Sputtering of ice by low-energy ions. *Surface Science* **2008**, *602*, 156–161.

(23) Gainullin, I. K. Resonant charge transfer during ion scattering on metallic surfaces. *Physics-Uspekhi* **2020**, *63*, 888–906.

(24) Buitrago, P. F.; Romero, M. A.; Bonin, C. J.; Irusta, Y.; González, C.; Vidal, R.; García, E. A.; Bonetto, F. Neutralization of low-energy Ne+ colliding with MoS_2 and metallic molybdenum: An experimental and theoretical study. *Physical Review A* **2024**, *110*.

(25) Hagstrum, H. D. Auger Electron Ejection from Germanium and Silicon by Noble Gas Ions. *Physical Review* **1960**, *119*, 940–952.

(26) Goedecker, S.; Teter, M.; Hutter, J. Separable dual-space Gaussian pseudopotentials. *Physical Review B* **1996**, *54*, 1703–1710.

(27) Hutter, J.; Iannuzzi, M.; Schiffmann, F.; VandeVondele, J. cp2k: atomistic simulations of condensed matter systems. *WIREs Computational Molecular Science* **2013**, *4*, 15–25.

(28) VandeVondele, J.; Krack, M.; Mohamed, F.; Parrinello, M.; Chassaing, T.; Hutter, J. Quickstep: Fast and accurate density functional calculations using a mixed Gaussian and plane waves approach. *Computer Physics Communications* **2005**, *167*, 103–128.

(29) Lippert, G.; Hutter, J.; Parrinello, M. A hybrid Gaussian and plane wave density functional scheme. *Molecular Physics* **1997**, *92*, 477–487.

(30) VandeVondele, J.; Hutter, J. An efficient orbital transformation method for electronic structure calculations. *The Journal of Chemical Physics* **2003**, *118*, 4365–4369.

(31) Kühne, T. D.; Krack, M.; Mohamed, F. R.; Parrinello, M. Efficient and Accurate Car-Parrinello-like Approach to Born-Oppenheimer Molecular Dynamics. *Physical Review Letters* **2007**, *98*.

(32) Kolafa, J. Time-reversible always stable predictor–corrector method for molecular dynamics of polarizable molecules. *Journal of Computational Chemistry* **2003**, *25*, 335–342.

(33) Weber, V.; VandeVondele, J.; Hutter, J.; Niklasson, A. M. N. Direct energy functional minimization under orthogonality constraints. *The Journal of Chemical Physics* **2008**, *128*.

(34) Hamilton, T. P.; Pulay, P. Direct inversion in the iterative subspace (DIIS) optimization of open-shell, excited-state, and small multiconfiguration SCF wave functions. *The Journal of Chemical Physics* **1986**, *84*, 5728–5734.

(35) Morozov, I. V.; Norman, G. E.; Valuev, A. A. Stochastic properties of strongly coupled plasmas. *Physical Review E* **2001**, *63*.

(36) Perdew, J. P.; Burke, K.; Ernzerhof, M. Generalized Gradient Approximation Made Simple. *Physical Review Letters* **1996**, *77*, 3865–3868.

(37) Furness, J. W.; Kaplan, A. D.; Ning, J.; Perdew, J. P.; Sun, J. Accurate and Numerically Efficient r2SCAN Meta-Generalized Gradient Approximation. *The Journal of Physical Chemistry Letters* **2020**, *11*, 8208–8215.

(38) Sabatini, R.; Gorni, T.; de Gironcoli, S. Nonlocal van der Waals density functional made simple and efficient. *Physical Review B* **2013**, *87*.

(39) Grimme, S.; Antony, J.; Ehrlich, S.; Krieg, H. A consistent and accurateab initioparametrization of density functional dispersion correction (DFT-D) for the 94 elements H-Pu. *The Journal of Chemical Physics* **2010**, *132*.

(40) Ma, Q.; Odenthal, P. M.; Mann, J.; Le, D.; Wang, C. S.; Zhu, Y.; Chen, T.; Sun, D.; Yamaguchi, K.; Tran, T. et al. Controlled argon beam-induced desulfurization of monolayer molybdenum disulfide. *Journal of Physics: Condensed Matter* **2013**, *25*, 252201.

(41) Xie, J.; Zhang, J.; Li, S.; Grote, F.; Zhang, X.; Zhang, H.; Wang, R.; Lei, Y.; Pan, B.; Xie, Y. Controllable Disorder Engineering in Oxygen-Incorporated MoS2 Ultrathin Nanosheets for Efficient Hydrogen Evolution. *Journal of the American Chemical Society* **2013**, *135*, 17881–17888.

(42) Ning, J.; Kothakonda, M.; Furness, J. W.; Kaplan, A. D.; Ehlert, S.; Brandenburg, J. G.; Perdew, J. P.; Sun, J. Workhorse minimally empirical dispersion-corrected density functional with tests for weakly bound systems: r2SCAN + rVV10. *Physical Review B* **2022**, *106*.

(43) consortium, T. P. Promoting transparency and reproducibility in enhanced molecular simulations. *Nature Methods* **2019**, *16*, 670–673.

(44) Tribello, G. A.; Bonomi, M.; Branduardi, D.; Camilloni, C.; Bussi, G. PLUMED 2: New feathers for an old bird. *Computer Physics Communications* **2014**, *185*, 604–613.

(45) Bonomi, M.; Branduardi, D.; Bussi, G.; Camilloni, C.; Provasi, D.; Raiteri, P.; Donadio, D.; Marinelli, F.; Pietrucci, F.; Broglia, R. A. et al. PLUMED: A portable plugin for free-energy calculations with molecular dynamics. *Computer Physics Communications* **2009**, *180*, 1961–1972.

(46) Barducci, A.; Bussi, G.; Parrinello, M. Well-Tempered Metadynamics: A Smoothly Converging and Tunable Free-Energy Method. *Physical Review Letters* **2008**, *100*.

(47) Laio, A.; Parrinello, M. Escaping free-energy minima. *Proceedings of the National Academy of Sciences* **2002**, *99*, 12562–12566.

(48) Peng, H.; Perdew, J. P. Rehabilitation of the Perdew-Burke-Ernzerhof generalized gradient approximation for layered materials. *Physical Review B* **2017**, *95*.

(49) Young, P. A. Lattice parameter measurements on molybdenum disulphide. *Journal of Physics D: Applied Physics* **1968**, *1*, 936–938.
