# Supplementary material for: Transition Metal Dichalcogenide MoS2: Oxygen and Fluorine Functionalization for Selective Plasma Processing
Source: J Phys Chem Lett. 2026 Apr 27;17(18):5207–14. doi: 10.1021/acs.jpclett.6c00348 (PMC13158984; doi:10.1021/acs.jpclett.6c00348)
Supplement: Supplementary file 2 [file jz6c00348_si_002.pdf]

jz-2026-003484.R1

Name: Peer Review Information for "Transition Metal Dichalcogenide MoS₂: oxygen and fluorine functionalization for selective plasma processing"

First Round of Reviewer Comments

Reviewer: 1

Comments to the Author

This manuscript shows the effect of O and F adsorption on the surface of MoS2 on Sulfur Selective Etching using ab-initio molecular dynamics (AIMD). They identified the mechanism of 'Chemically Enhanced Physical Sputtering' that induces the formation of volatile species such as SO2 and SFx on the surface of MoS2O and MoS2F where O and F are adsorbed to MoS2. Through this, they showed that the sulfur sputtering critical energy can be reduced from the existing 31 eV to the level of 10 eV, and the process margin (window) can be secured without damaging the metal lattice. These results are interesting and I think that this manuscript shows some important aspects to the researchers working on MoS2 etching. On the other hand, there are some parts that are not clear, and if the answers to the comments presented below are included in the paper, it is judged that the above paper can be publishable in the Journal of physical chemistry letter.

1. Evaluating the impact on different adsorption sites

This study dealt with the model of O or F attaching to S, but in the real world, it is likely to bind directly to Mo or be replaced by an already existing S-Vacancy. Further confirmation is needed to find out if these different adsorption structures can reduce selectivity or affect critical energy, such as the Chlorine adsorption study of MoS2 (DOI: 10.1039/c9tc05548 g).

2. Basis for adsorption coverage value

In the manuscript, coverage 10% was set as the threshold for mechanism operation, but what is the basis for this specific value?

3. Consideration of residual atoms and surface conditions after sputtering,

It needs to be shown if the remaining O or F on the surface is completely desorbed after sulfur is removed in the form of SO_2 or SF_x using the proposed critical energy. If it remains on the surface, it may affect the characteristics of the device, and additional processes will need to be taken to remove it.

4. Spontaneous etching in actual processes

A reference paper (DOI:10.1002/admi.202000762) reported that etching occurs only by the chemical reaction between F Ion/Radical and MoS_2 in an SF_6 plasma system. Therefore, further examination is needed to determine whether the self-limiting properties of the S atom can be maintained even under the high reactivity conditions of the SF_6 gas (F).

Reviewer: 2

Comments to the Author

From an experimental point of view, this is a very interesting paper which provides insights into the sputtering processes that can take place on TMD surfaces when they are exposed to Ar plasmas. which is relevant for plasma cleaning, plasma etching and plasma deposition on TMD surfaces. The paper is accessible to experimentalists, which increases the impact of the paper.

There are a few things I would suggest to add to the paper:

1) I cannot judge how much work this is, but adding TMD hydrogen termination to the simulations would be very valuable: H_2 plasmas are often used to clean TMDs and hydrogen containing plasmas are also used in PEALD of TMDs. Both are often done in combination with Ar. Hydrogen containing plasmas will lead to hydrogen termination, which most likely will affect the impact of Ar as well. It would be really interesting to see how H termination impacts Ar sputter behaviour.

2) I would like to see a discussion on how this work can be extended to other TMD materials.

Minor suggestions:

1) Please describe what the dotted lines figure 5b represent.

Author's Response to Peer Review Comments:

Re: jz-2026-003484

Dear Editor,

We appreciate the positive and constructive comments from the Referees. We are submitting a revised version of our manuscript, where we addressed all the changes/questions suggested by the referees. Additions to the main text are marked in blue in the file main_colored.pdf. Please find below the response to each of the Referee's questions/concerns, along with a list of modifications in the revised manuscript (response_ jz-2026-003484_Polyachenko.docx).

Sincerely,
Yury Polyachenko, Yuri Barsukov, Shoaib Khalid, Igor Kaganovich

Reply to the First Referee - jz-2026-003484

Referee: Evaluating the impact on different adsorption sites
This study dealt with the model of O or F attaching to S, but in the real world, it is likely to bind directly to Mo or be replaced by an already existing S-Vacancy. Further confirmation is needed to find out if these different adsorption structures can reduce selectivity or affect critical energy, such as the Chlorine adsorption study of MoS2 (DOI: 10.1039/c9tc05548g).

Response:

We thank the referee for raising this important point regarding alternative adsorption configurations. We acknowledge that binding of reactive species directly to Mo sites or at S vacancies has been reported in the literature. However, we expect such configurations to be limited under the processing conditions considered in this work. In particular, previously reported energy barriers for direct Mo binding suggest that these processes are kinetically unfavorable [doi:10.1038/s41557-018-0136-2, 10.1021/nn500532f], allowing for a processing regime in which adsorption predominantly occurs on S sites

Furthermore, the conditions explored in this study correspond to the onset of damage (i.e., low-energy sputtering thresholds), where the defect concentration is expected to remain minimal. Since Mo site binding is often facilitated by the presence of defects such as S vacancies [doi:10.1063/1.4916536], the low-defect-density regime considered here further reduces the likelihood of such adsorption pathways.

In addition, S-O substitution in defect-free MoS_2 has a relatively high activation barrier (~1.1 eV, Refs. 10.1038/s41557-018-0136-2 and 10.1021/nn500532f), corresponding to long

timescales (1-10 months range) at near ambient conditions. Such processes can therefore be effectively suppressed by short exposure times (e.g., < 1 minute).

Referee: Basis for adsorption coverage value
In the manuscript, coverage 10% was set as the threshold for mechanism operation, but what is the basis for this specific value?

Response:

We thank the referee for this important question. The value of 10% coverage is not intended as a strict threshold, but rather as a representative value indicating when the enhancement mechanism becomes significantly suppressed.

The chemically enhanced sputtering pathway relies on the formation of volatile species (e.g., SO2), which requires adjacent S sites to be occupied by adsorbates on the MoS2 lattice. Therefore, the probability of activating this pathway depends on the likelihood of finding neighboring occupied sites. Based on simple combinatorial arguments (see revised SI, Section "Surface coverage fraction effects", Eq. S2), this probability scales approximately linearly with the coverage fraction ($0 < c < 1$).

As a result, at $c = 0.1$ (10% coverage), the probability of activating the enhanced pathway is estimated to be reduced by about an order of magnitude compared to full coverage ($c = 1.0$). We therefore use 10% as a physically meaningful reference point to indicate when the mechanism becomes substantially less effective, rather than as a strict cutoff. This clarification has been added to the revised manuscript on page 7(see below) and Supporting Information.

"Full surface coverage is considered in our model. The reduction in the sputtering threshold arises from the formation and desorption of products such as SO2 and SF4. Thus, using combinatorial arguments about neighboring sites occupation by oxygen or fluorine, we show that surface coverage $c \in (0;1)$ should decrease the probability of the proposed mechanisms by c (details in SI ``Surface coverage fraction effects'')"

Referee: Consideration of residual atoms and surface conditions after sputtering
It needs to be shown if the remaining O or F on the surface is completely desorbed after sulfur is removed in the form of SO2 or SFx using the proposed critical energy. If it remains on the surface, it may affect the characteristics of the device, and additional processes will need to be taken to remove it.

Response:

We thank the referee for highlighting this important point. We agree that the fate of residual O or F after sputtering and the subsequent surface cleaning is critical for device applications.

In the present work, our primary focus is on the sputtering mechanism and the reduction of critical energy. The removal of remaining functionalizing species is a separate and non-trivial step that depends on post-processing conditions. Previous studies suggest that chemisorbed species can be removed through approaches such as high-vacuum annealing or exposure to reactive species (e.g., atomic hydrogen), which have been shown to be effective in cleaning oxidized metal surfaces. For example, exposure to atomic hydrogen has been used to remove oxygen from d-metal surfaces such as Mo [Tsarfati et al. "Atomic O and H exposure of C-covered and oxidized d-metal surfaces", Surface Science 603, 2594 (2009), doi:10.1016/j.susc.2009.06.008]. It is expected that the resulting clean d-metal surface exhibits a very high recombination coefficient for H-atoms [Kitajima et al. Journal of Nuclear Materials 141-143, 234 (1986), doi:10.1016/0022-3115(86)90107-8]. There is also a brief discussion about cleaning chemisorbed O atoms from MoS2 via high vacuum (Ref. 52 in main text)

We have added a brief note in the manuscript on Page 16(see below) to clarify that post-etch cleaning is necessary and that a systematic investigation of suitable cleaning strategies is currently underway and will be addressed in future work.

"Cleaning the functionalizing O/F atoms after achieving the desired processing effect also requires additional steps such as high-vacuum annealing [10.1038/srep11921]. The removal of residual functionalizing atoms is essential to preserve downstream device quality, motivating further systematic studies of post-processing cleaning approaches."

Referee: Spontaneous etching in actual processes

A reference paper (DOI:10.1002/admi.202000762) reported that etching occurs only by the chemical reaction between F Ion/Radical and MoS2 in an SF6 plasma system. Therefore, further examination is needed to determine whether the self-limiting properties of the S atom can be maintained even under the high reactivity conditions of the SF6 gas (F).
Response:

The referee raises an important point regarding the behavior under highly reactive SF6 plasma conditions. We agree that purely chemical etching by F radicals can occur, as demonstrated in the cited work. However, the present study focuses on Ar plasma conditions, where chemical reactivity is minimal and the mechanism is dominated by ion-induced processes. In this regime, we do not expect significant spontaneous chemical etching driven by neutral species alone.

In the cited paper, SF6 plasma was used for TMD etching. We believe that there reactive F^+ ions are essential for TMD etching and F sputtering threshold is below the Ar sputtering threshold. By contrast, a plasmaless treatment at low temperature may enable fluorine termination of the sulfur surface without causing etching. Indeed, *Farigliano et al.* ("Ab-initio molecular dynamics simulations of the reactivity of MoS_2 towards F_2 molecules: Implications for etching processes," *Applied Surface Science* 607 (2023) 154637, doi:10.1016/j.apsusc.2022.154637) showed using *ab initio* molecular dynamics that *The*

dissociative adsorption of F2 occurs at very low temperatures (100 K) and the surface becomes populated by SF, SF2 (the most stable), and SF3 groups. Etching processes begin with the desorption of the SF3 group (at 500+ K) and only at very high temperatures the desorption of SF2 was observed.

We also note that studies using similarly reactive fluorine-containing precursors (e.g., MoF6, Ref. 10.1021/acs.chemmater.2c02549) show that fluorination of MoS2 does not by itself lead to continuous etching. Instead, etching proceeds only when a second reactant (e.g., H2O) is introduced to complete the reaction cycle in an atomic layer etching scheme. This suggests that sustained chemical etching requires synergistic alternations rather than occurring spontaneously under a single-species treatment.

Furthermore, under near-ambient, (i.e., without plasma) conditions, we do not expect F from MoS2F to desorb due to a relatively high barrier (~1.3 eV, Fig. S3), indicating that the fluorinated surface remains stable in the absence of energetic activation.

We have added a discussion in the revised manuscript on page 16(see below) to clarify that while SF6 based plasmas may lead to chemically driven etching, the self-limiting behavior identified here is expected to hold under inert (e.g., Ar) plasma conditions, and that extension to highly reactive environments requires further dedicated investigation.

"Additionally, the stability of the system in the presence of chemically reactive gases, such as \ce{SF6} used during functionalization, warrants further investigation. Notably, a similarly reactive gas, \ce{MoF6}, has been employed in atomic-layer etching \cite{Soares2023} and was observed to induce damage only when alternated with another reactant, such as \ce{H2O}"

Reply to the Second Referee - jz-2026-003484

Referee: There are a few things I would suggest to add to the paper:
I cannot judge how much work this is, but adding TMD hydrogen termination to the simulations would be very valuable: H2 plasmas are often used to clean TMDs and hydrogen containing plasmas are also used in PEALD of TMDs. Both are often done in combination with Ar. Hydrogen containing plasmas will lead to hydrogen termination, which most likely will affect the impact of Ar as well. It would be really interesting to see how H termination impacts Ar sputter behaviour.

Response:

We thank the referee for raising an interesting and important point regarding hydrogen termination. We agree that hydrogen-containing plasmas are highly relevant in practical processing and may influence sputtering behavior.

In the present work, we focused on Ar-only plasma conditions to isolate the role of ion-induced sputtering while minimizing additional chemical effects. Previous studies [E. W. Keong Koh *et al Int. J. Hydrog. Energy.* 37 14323-14328 (2012), doi:10.1016/j.ijhydene.2012.07.069; S Khalid *et al 2D Mater.* 11 031003 (2024), doi:10.1088/2053-1583/ad4720] have shown that hydrogen can adsorb at multiple sites in MoS2, including an interstitial site in the plane of the metal(Mo) atom inside the hexagonal lattice. Thus, a quantitative investigation of hydrogen termination would require a separate, systematic study, including adsorption energetics, equilibrium structure determination, and extensive AIMD sampling over impact energies and angles for each of the adsorption sites, which is beyond the scope of the current work.

Nevertheless, we expect that principles of the chemically enhanced sputtering mechanisms identified in this work are generalizable. Specifically, similar chemically enhanced sputtering could occur in the presence of hydrogen. In particular, the formation of volatile species such as H2S may facilitate sulfur removal, similar to the SO2 and SF_n pathways identified here. However, due to the lower mass of hydrogen, the efficiency of kinetic energy transfer from Ar is reduced, which may result in a higher threshold energy compared to O-functionalized systems.

We have added a brief discussion in the manuscript on Page 14(see below) to highlight the potential role of hydrogen termination and to indicate this as an important direction for future work.

"A similar argument is expected to hold for most functionalizing atoms, except for very light species such as hydrogen. Because hydrogen is much lighter, it is less effective at slowing down incoming Ar atoms, so the resulting collision pathways are likely to differ from those involving O or F. In addition, hydrogen adsorption is more complex, likely due to its small size. Atomic hydrogen can occupy several competing adsorption sites, including an interstitial site in the plane of the metal(Mo) atom inside the hexagonal lattice \cite{KeongKoh2012}. Consequently, more detailed studies are required to quantitatively assess sputtering in H functionalized TMDs such as \ce{MoS2H}"

Referee: I would like to see a discussion on how this work can be extended to other TMD materials.

<u>Response:</u>

This is a very helpful suggestion. We agree that extending these insights to other TMD materials is an important direction. We conducted preliminary AIMD simulations for MoSe2, WS2 and WSe2 terminated with O and F and have added a short discussion on page 14(see below) describing how the mechanisms identified here may apply more broadly to other TMD systems.

“The main mechanism responsible for the reduction in energy threshold is attributed to the formation and subsequent desorption of products such as \ce{SO2} and \ce{SF_n}. A fully quantitative investigation is required to establish the limits of generalization of this mechanism; however, we expect similar behavior to occur in other TMDs and for other functionalizations. Preliminary AIMD simulations for \ce{MoSe2}, \ce{WS2}, and \ce{WSe2} with 100\% O and F termination indicate that the formation of species such as \ce{SF3}, \ce{SO2}, \ce{SeF3}, and \ce{SeO2} dominates the sputtering pathways near the threshold energies. Functionalization with O or F similarly reduces the sputtering threshold by approximately a factor of three, from $\sim$30 eV to $\sim$10 eV. In the case of oxygen termination, the hexagonal structure is largely preserved, and the system retains sensitivity to the impact direction. In contrast, fluorine termination breaks the hexagonal symmetry, as observed for \ce{MoS2F}, and the directional dependence is largely lost. Substitution of \ce{W} for \ce{Mo} does not lead to significant differences in the threshold energy within the uncertainty of $\pm$3 eV.”

Minor suggestions:
Referee: Please describe what the dotted lines figure 5b represent.

Response:

We appreciate the referee for pointing out this lack of clarity. The dotted lines in Fig. 5b represent the confidence intervals derived from the error bars shown in Fig. 4. This has now been clarified in the figure caption.

List of Modifications:

1) **Added the following lines on Page 7-8:**

   “Full surface coverage is considered in our model. The reduction in the sputtering threshold arises from the formation and desorption of products such as \ce{SO2} and \ce{SF4}. Thus, using combinatorial arguments about neighboring sites occupation by oxygen or fluorine, we show that surface coverage $c \in (0;1)$ should decrease the probability of the proposed mechanisms by c (details in SI ``Surface coverage fraction effects')”

2) **Added the following on Page 11 (expanding the chemical intuition behind the differences in F vs O adsorbed layer structures):**

   “We believe this is analogous to ``Peierls distortions'' \cite{Kagoshima1981, Peierls2001, Burdett1983, You2021} due to odd number of electrons brought to a unit cell by a fluorine atom (see SI for more discussion)”

3) **Added the following lines on Page 16:**

“The removal of residual functionalizing atoms is essential to preserve downstream device quality, motivating further systematic studies of post-processing cleaning approaches. Additionally, the stability of the system in the presence of chemically reactive gases, such as \ce{SF6} used during functionalization, warrants further investigation. Notably, a similarly reactive gas, \ce{MoF6}, has been employed in atomic-layer etching \cite{Soares2023} and was observed to induce damage only when alternated with another reactant, such as \ce{H2O}.”

4) Added the following lines on Page 14-15:

“A similar argument is expected to hold for most functionalizing atoms, except for very light species such as hydrogen. Because hydrogen is much lighter, it is less effective at slowing down incoming Ar atoms, so the resulting collision pathways are likely to differ from those involving O or F. In addition, hydrogen adsorption is more complex, likely due to its small size. Atomic hydrogen can occupy several competing adsorption sites, including an interstitial site in the plane of the metal (Mo) atom inside the hexagonal lattice \cite{KeongKoh2012,khalid2024Role}. Consequently, more detailed studies are required to quantitatively assess sputtering in H functionalized TMDs such as \ce{MoS2H}.”

***5)* Added the following lines on Page 14:**

“The main mechanism responsible for the reduction in the energy threshold is attributed to the formation and subsequent desorption of products such as \ce{SO2} and \ce{SF_n}. A fully quantitative investigation is required to establish the limits of generalization of this mechanism; however, we expect similar behavior to occur in other TMDs and for other functionalizations. Preliminary AIMD simulations for \ce{MoSe2}, \ce{WS2}, and \ce{WSe2} with 100\% O and F termination indicate that the formation of species such as \ce{SF3}, \ce{SO2}, \ce{SeF3}, and \ce{SeO2} dominates the sputtering pathways near the threshold energies. Functionalization with O or F similarly reduces the sputtering threshold by approximately a factor of three, from $\sim$30 eV to $\sim$10 eV. In the case of oxygen termination, the hexagonal structure is largely preserved and the system retains sensitivity to the impact direction. In contrast, fluorine termination breaks the hexagonal symmetry, as observed for \ce{MoS2F}, and the directional dependence is largely lost. Substitution of \ce{W} for \ce{Mo} does not lead to significant differences in the threshold energy within the uncertainty of $\pm$3 eV.”

6) Moved the figure that was last in the SI to Fig.5(a)

7) Updated captions in Fig 5:

(a): AIMD simulation threshold (datapoints) at different temperatures for $\theta = 0$ and their predictions (dashed: mean, dotted: confidence interval) based on $\EArPerp(T)$ from eq.\eqref{eq:mainTh}. Red and green correspond to \ce{MoS2O} and \ce{MoS2F} respectively. No fitting parameters were used (with the exception of the spline parameters used for interpolating spline data of $\EArX(\theta)$ from Figure \ref{fig:F4}). (a-inset): A

schematic of an \ce{Ar} impacting an O atom (red solid circle) that thermally fluctuated from its equilibrium position (red dashed circle). The \ce{Ar} velocity is directed exactly at the O equilibrium position, which was shown to be the most damage-susceptible point of \ce{MoS2O} in Figure \ref{fig:F2S5}. However, the impact is not head-on due to a thermal fluctuation of magnitude $\sigma(T)$ of the O atom.

…….Dotted lines in (a) and (b) show confidence intervals based on error bars in Figure 4.

8) **Added Ref 13, 59, 60, 61, 62, 65, 66, 70**

9) **Added “Sputtering yield at near-threshold energies” section to the SI**
10) **Added “Surface coverage fraction effects” section to the SI**
11) **Added a paragraph on “Peierls distortions” in the section “Sputtering mechanism differences for MoS2O vs MoS2F” of the SI**
12) **Added a paragraph on the collision event durations less than the “dynamical memory” time in the “DFT and AIMD details” section. It ensures the AIMD method is applicable not only for sampling, but for dynamical trajectories resolution, which is necessary for our results to be valid.**
13) **Added SI references covering the new sections and paragraphs**
